# Peiminine Reduces ARTS-Mediated Degradation of XIAP by Modulating the PINK1/Parkin Pathway to Ameliorate 6-Hydroxydopamine Toxicity and α-Synuclein Accumulation in Parkinson’s Disease Models In Vivo and In Vitro

**DOI:** 10.3390/ijms221910240

**Published:** 2021-09-23

**Authors:** Yu-Ling Hsu, Huey-Shan Hung, Chia-Wen Tsai, Shih-Ping Liu, Yu-Ting Chiang, Yun-Hua Kuo, Woei-Cherng Shyu, Shinn-Zong Lin, Ru-Huei Fu

**Affiliations:** 1 Graduate Institute of Biomedical Sciences, China Medical University, Taichung 40402, Taiwan; fish38@cgmh.org.tw (Y.-L.H.); hunghs@mail.cmu.edu.tw (H.-S.H.); spliu@mail.cmu.edu.tw (S.-P.L.); tina.chiang831@gmail.com (Y.-T.C.); shyu9423@gmail.com (W.-C.S.); 2 Translational Medicine Research Center, China Medical University Hospital, Taichung 40447, Taiwan; 3 Department of Nutrition, China Medical University, Taichung 40402, Taiwan; cwtsai@mail.cmu.edu.tw; 4 Department of Nursing, Taipei Veterans General Hospital, Taipei 12217, Taiwan; yhkuo3@vghtpe.gov.tw; 5 Bioinnovation Center, Tzu Chi Foundation, Department of Neurosurgery, Buddhist Tzu Chi General Hospital, Tzu Chi University, Hualien 970, Taiwan; shinnzong@yahoo.com.tw; 6 Department of Psychology, Asia University, Taichung 41354, Taiwan

**Keywords:** peiminine, Parkinson’s disease, 6-hydroxydopamine, α-synuclein, apoptosis, proteasome, autophagy, parkin, ARTS, XIAP

## Abstract

Parkinson’s disease (PD) is a degenerative disease that can cause motor, cognitive, and behavioral disorders. The treatment strategies being developed are based on the typical pathologic features of PD, including the death of dopaminergic (DA) neurons in the substantia nigra of the midbrain and the accumulation of α-synuclein in neurons. Peiminine (PMN) is an extract of *Fritillaria thunbergii* Miq that has antioxidant and anti-neuroinflammatory effects. We used *Caenorhabditis elegans* and SH-SY5Y cell models of PD to evaluate the neuroprotective potential of PMN and address its corresponding mechanism of action. We found that pretreatment with PMN reduced *reactive oxygen species* production and DA neuron degeneration caused by exposure to 6-hydroxydopamine (6-OHDA), and therefore significantly improved the DA-mediated food-sensing behavior of 6-OHDA-exposed worms and prolonged their lifespan. PMN also diminished the accumulation of α-synuclein in transgenic worms and transfected cells. In our study of the mechanism of action, we found that PMN lessened ARTS-mediated degradation of X-linked inhibitor of apoptosis (XIAP) by enhancing the expression of PINK1/parkin. This led to reduced 6-OHDA-induced apoptosis, enhanced activity of the ubiquitin–proteasome system, and increased autophagy, which diminished the accumulation of α-synuclein. The use of small interfering RNA to down-regulate parkin reversed the benefits of PMN in the PD models. Our findings suggest PMN as a candidate compound worthy of further evaluation for the treatment of PD.

## 1. Introduction

Parkinson disease (PD) is a degenerative disease of dopamine neurons caused by environmental or genetic factors that most often occurs in adults older than 60 years. There are currently about 10 million patients with PD worldwide, and the corresponding impacts on the family, economy and medical system are considerable [1]. The detailed pathologic mechanism of PD is still unclear. Loss of dopaminergic (DA) neurons in the substantia nigra compact area of the midbrain is a typical pathologic feature of PD, which results in a lack of dopamine in the basal ganglia and eventually leads to the onset of clinical symptoms, such as bradykinesia, rigidity, tremors, unstable posture, and cognitive and behavioral problems [2].

Another common and important feature of PD pathology is the accumulation of insoluble cytoplasmic α-synuclein, and the formation of Lewy bodies and Lewy neurites in neurons. Therefore, PD is also known as a synucleinopathy [3]. α-Synuclein contains 140 amino acids and has two forms: free and membrane-bound. It is encoded by the SNCA gene and occupies about 1% of the cytoplasmic protein of brain cells, partly in the mitochondria and nucleus. The main function of α-synuclein may be to regulate the activity of synaptic vesicles, the release of neurotransmitters, and the recycling of synaptic vesicles. In addition, studies have shown that α-synuclein is involved in neuronal Golgi and vesicle transport, membrane structure regulation, lipid metabolism, mitochondrial fusion, DNA repair, and cognitive functions [4]. The structure of α-synuclein is unstable, and the α-helix conformer is easily replaced by a β-sheet. This replacement results in misfolding and aggregation of α-synuclein to form oligomers or fibrils, which may be related to sporadic PD, with the end result being dysregulation of the synaptic, mitochondrial, and protein homeostasis systems; oxidative stress; microtubule damage; and abnormal calcium signaling [5].

Mutants of α-syncline have been found to be related to familial PD. These include A53T, A30P, E46K, H50Q, and G51D [6]. Overexpression of human wild-type or A53T mutant α-synuclein in animals can lead to the deposition of α-synuclein in brain neurons, causing neurodegenerative toxicity and damage to the dopaminergic system and movement function, and thus can be used as a PD model [7].

The oxidative stress caused by mitochondrial damage, respiratory chain obstruction, and antioxidant enzyme defects reflects the imbalance between the generation of reactive oxygen species (ROS) and the efficiency of cells to detoxify or repair reactive oxygen intermediates, which ultimately leads to protein, lipid, and DNA damage and disturbance of redox signals in cells. These are closely related to the degeneration of DA neurons [8]. One report indicated that excessive ROS in the substantia nigra compact lead to apoptosis of DA neurons [9]. The neurotoxic compound 6-hydroxydopamine (6-OHDA) can enter cells through DA reuptake transporters and produce ROS, which in turn can selectively destroy DA neurons by inducing apoptosis, reducing proteasome activity, and preventing autophagy. 6-OHDA has thus been widely used in various pharmacologic models of PD.

In recent years, important clinical and basic studies have shown that two mitochondrial quality-control mechanisms, autophagy and the ubiquitin-proteasome system (UPS), can help cells resist various types of cellular stress through the PINK1/parkin pathway. The inner mitochondrial membrane protein PINK1 (PTEN-induced kinase 1) is a serine/threonine kinase. Ubiquitin E3 ligase parkin can add ubiquitin to a target protein to promote degradation of the protein by the 26S proteasome. Many proteins are substrates of parkin, such as α-synuclein. It is known that some patients with autosomal-recessive PD have mutations in PINK1 and parkin [10]. 

In damaged mitochondria, PINK1 accumulates on the outer membrane and then recruits and activates parkin through the phosphorylation of the ubiquitin chain, which causes the degradation of mitochondria through autophagy or mitophagy or degradation of the target protein through the UPS [11,12]. Furthermore, reducing mitochondrial-dependent and mitochondrial-independent apoptosis to increase cell survival can also be achieved by regulating the activity of PINK1 and parkin [13,14].

X-linked inhibitor of apoptosis protein (XIAP), which belongs to the inhibitor of apoptosis family of proteins (IAP), can directly prevent the apoptotic activity of caspase through three baculoviral IAP repeats (BIR domains) [15]. XIAP also has a RING domain, which can act as an ubiquitin E3 ligase to catalyze the ubiquitination of specific substrate proteins and promote their degradation by the proteasome [16]. Moreover, some reports indicate that XIAP can upregulate autophagy [17].

Another protein of interest is ARTS (apoptosis-related protein in the TGF-β signaling pathway). ARTS, which is located in the outer membrane of mitochondria, is a pro-apoptotic protein. Once stimulated by the apoptotic signal, ARTS quickly translocates to the cytoplasm and binds to XIAP. The antagonized XIAP causes activation of caspase 9, 7, and 3, which leads to apoptosis [18]. ARTS can also act as a modulator of autophagy. Overexpression of ARTS leads to down-regulated autophagy and a lessened capacity for proliferation and migration in vascular smooth muscle cells [19]. Interestingly, ARTS has been proven to be a substrate of parkin. Parkin can reduce neuronal cell apoptosis by promoting the ubiquitination and degradation of ARTS [20]. Some PD patients with mutations of parkin may have augmented neuronal cell apoptosis caused by increased ARTS.

**Figure 1:**
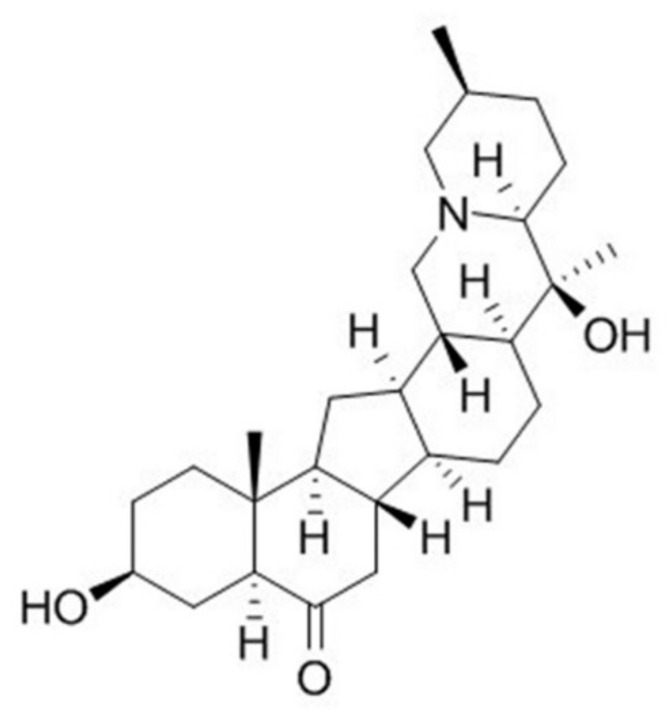
Chemical structure of peiminine (PMN).

At present, in addition to treatments for alleviating the symptoms of PD, there are no successful treatments to effectively slow down or improve the progress of PD. The aglycone alkaloid peiminine (PMN, Figure 1), which is isolated from the bulbs of *Fritillaria thunbergii* Miq, is a traditional herbal medicine of Southeast Asia, namely zhe bei mu. PMN has been confirmed to have several pharmacologic effects linked to anti-inflammatory and antioxidant activity [21]. For example, Du et al. showed that PMN reduces acute lung injury caused by lipopolysaccharide (LPS) in mice by inhibiting inflammation-related factors and the formation of lipid rafts [22]. Luo et al. indicated that PMN can improve interleukin-1β (IL-1β)-induced osteoarthritis in mice by inhibiting the inflammatory response of chondrocytes [23]. Chen et al. revealed that PMN can prevent neuroinflammation and protect DA neurons in the LPS-induced PD model in rat [24]. However, the effectiveness of PMN against PD-related oxidative stress and α-synuclein accumulation has not been evaluated.

In the present study, we first used *Caenorhabditis elegans* as an in vivo model of PD and a platform for evaluating the neuroprotective potential of PMN, because it has DA neurons, humans PD-related orthologous genes, known dopamine-regulated behavior patterns, easy-to-obtain genetically modified strains, low cost, short life cycle, transparent body and other advantages [25,26,27]. Primary mammalian neurons are terminal mature cells, which cannot be propagated in vitro and have limited use. The use of transformed neuronlike cells can overcome this limitation. The SH-SY5Y neuroblastoma cell line was derived from a metastatic bone tumor biopsy. It expresses tyrosine hydroxylase (TH) and dopamine-β-hydroxylase, which are characteristic of catecholaminergic neurons, and can be differentiated to a more mature neuronlike phenotype by treatment with retinoic acid. Thus, we used the SH-SY5Y cell line as in vitro model to further confirm the neuroprotective ability of PMN and explore its anti-parkinsonism mechanism [28,29].

## 2. Results

### 2.1. Toxicity of Peiminine in Worms

**Figure 2:**
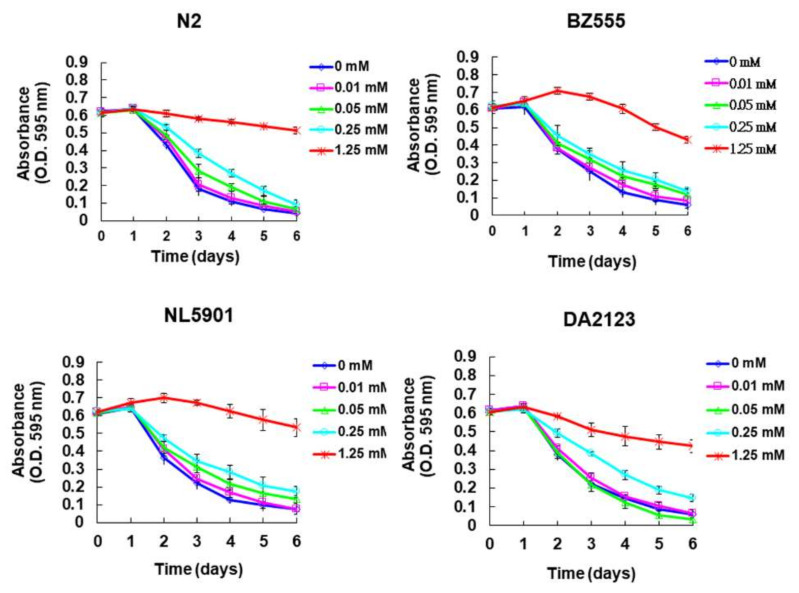
Evaluation of the toxicity of peiminine (PMN) in worms by food clearance test. In 96-well plates, L1 stage worms of four strain were cultured on OP50 *E. coli* (OD A_595_ = 0.6) feeding S-medium containing four concentrations of PMN, respectively. Cultivation was continued for 6 days, and the OD value of each group was measured and recorded daily.

The toxicity of PMN in the worm models was evaluated by use of food clearance tests. Compared with the food clearance curve in the untreated group of worms, the curve of the N2, BZ555, NL5901, and DA2123 strains was significantly slowed when 1.25 mM PMN was added to the culture (Figure 2). Observation with a dissecting microscope showed that the offspring decreased in number and body size (data not shown), which reflects the toxicity of PMN and a significant reduction in *E. coli* consumption. A PMN concentration of 0.25 mM did not significantly affect the food clearance curve of any strain of worms. Therefore, in subsequent experiments, the PMN concentration used to treat worms was set to a maximum of 0.25 mM.

### 2.2. PMN Pretreatment Significantly Reduces Dopaminergic Neuron Degeneration of 6-Hydroxydopamine-Exposed BZ555 Worms

**Figure 3:**
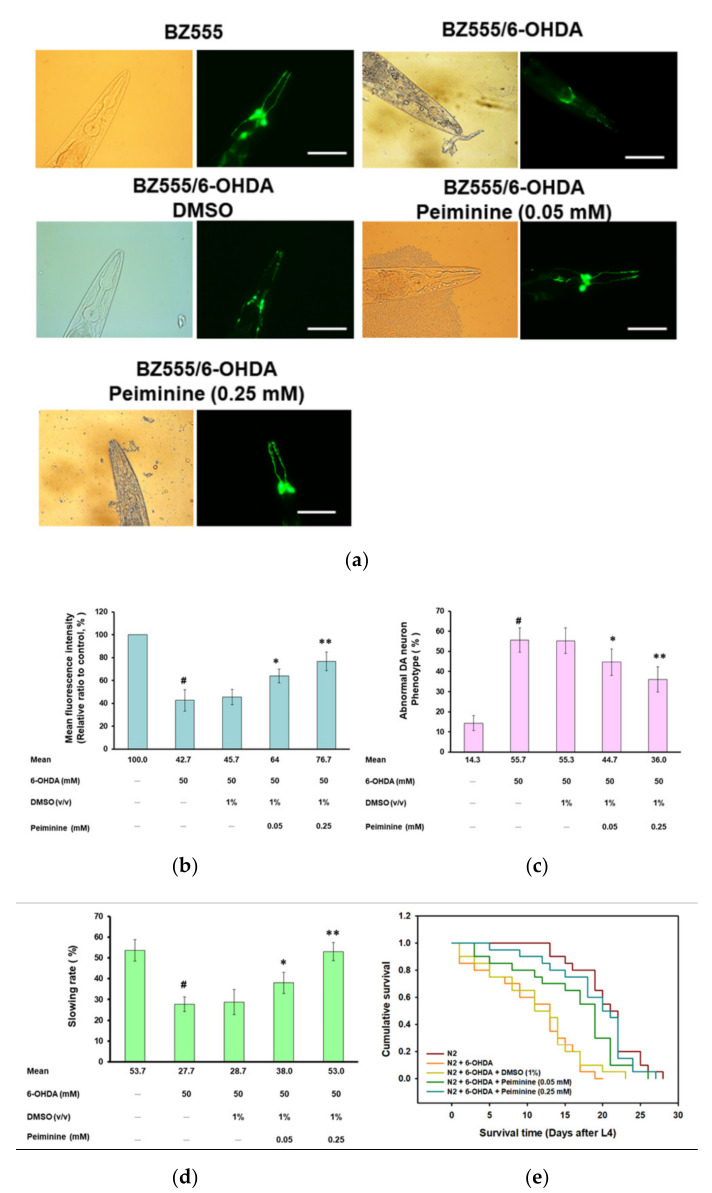
Dopaminergic (DA) neurons degeneration, food-sensing behavior defects, and shortened lifespan of worms caused by 6-hydroxydopamine (6-OHDA) are improved by peiminine (PMN) pretreatment. L1 stage worms were transferred to OP50/NGM plates with or without PMN and grown to the L3 stage, exposed to 6-OHDA for 1 h, and then transferred to OP50/NGM/FUDR plates with or without PMN and cultured for another 3 days before analysis. (**a**) Representative fluorescence images of GFP expression patterns in head DA neurons of BZ555 worms in each group. Scale bar = 50 µm. (**b**) The fluorescence intensity of the GFP image of each group was quantified in (**a**) using ImageJ software. (**c**) The DA neuron degeneration phenotype defects of each group in (**a**) were scored. The data are represented as a percentage of the total population of worms with defective DA neuron phenotypes. (**d**) Analysis of the food-sensing behavior of N2 worms in each group. The slowing rate was defined as the percentage decrease in the body bending frequency of worms on the lawn with bacteria compared with without bacteria (20 s on each type of lawn). (**e**) Cumulative survival curves of N2 worms in each group. In the above experiments, a total of 50 worms were counted in each group. ^#^ Indicates a significant difference between 6-OHDA-exposed and control worms (^#^ *p* < 0.001); * Indicates a significant difference between the PMN-pretreated, 6-OHDA-exposed and PMN-untreated, 6-OHDA-exposed groups (* *p* < 0.05, ** *p* < 0.01).

Fluorescence microscopy analysis showed that the GFP expression in the three pairs of DA neurons in the head of 6-OHDA-exposed BZ555 worms was significantly decreased, reflecting the destruction of DA neuron integrity (Figure 3a). PMN pretreatment significantly enhanced the GFP signals (Figure 3a). Using ImageJ software to quantify the fluorescence intensity, we found that the average GFP fluorescence intensity of 6-OHDA-exposed worms was lessened by 57.3% (*p* < 0.001) compared with that in control worms (Figure 3b). PMN pretreatment increased the fluorescence intensity of GFP in 6-OHDA-exposed worms in a dose-dependent manner. The 0.25 mm concentration of PMN raised the fluorescence intensity of DA neurons by 1.8-fold (*p* < 0.01) (Figure 3b).

Furthermore, the percentage of abnormal phenotypes in 6-OHDA-exposed worms was significantly higher than in control worms by 3.9-fold (*p* < 0.001) (Figure 3c). Compared with that in the 6-OHDA-exposed worms, the phenotype of DA neuron degeneration was significantly reduced in 6-OHDA-exposed worms pretreated with 0.25 mM PMN by 35.3% (*p* < 0.01) (Figure 3c).

### 2.3. Food-Sensing Behavioral Defects of 6-OHDA-Exposed Worms Are Restored by PMN Pretreatment

The DA neuron function of worms is reflected in food-sensing behavior. When in contact with food, worms reduce their speed of movement (the frequency of body bending) to increase the efficiency of eating. The results showed that, compared with that on nonbacterial lawns, the bending frequency of wild-type N2 worms decreased by 53.7% (*p* < 0.001) after contact with bacterial lawns (quantified by the “slowing rate”) (Figure 3d). Compared with the control group, 6-OHDA-exposed N2 worms displayed a significant lessening in the slowing rate by 48.4% (*p* < 0.001). PMN dose-dependently increased the slowing rate of worms exposed to 6-OHDA. Compared with the 6-OHDA-exposed worms, the slowing rate of worms pretreated with 0.25 mM PMN increased by 1.9-fold (*p* < 0.01) (Figure 3d). The above results confirm that the damage to DA neurons caused by 6-OHDA can be improved by PMN pretreatment.

### 2.4. Lifespan of 6-OHDA-Exposed Worms Is Extended by PMN Pretreatment

Patients with PD have a shortened lifespan. As shown in Figure 3e, the lifespan of 6-OHDA-exposed N2 worms was shorter than that of control worms. However, PMN pretreatment could dose-dependently prolong the lifespan of 6-OHDA-exposed worms (Figure 3e). The cumulative survival model of lifespan calculated using the Kaplan–Meier method showed that the average survival time of the 6-OHDA-exposed group was 11.8 ± 1.2 days, while the average survival time of the 0.25 mM PMN-pretreated 6-OHDA-exposed group was 19.8 ± 2.1 days (*p* < 0.001) (Figure 3e). Thus, the shortening of lifespan caused by 6-OHDA was improved by PMN pretreatment.

### 2.5. Accumulation of Human α-Synuclein in Muscle Cells of NL5901 Worms Is Reduced by PMN Treatment

**Figure 4:**
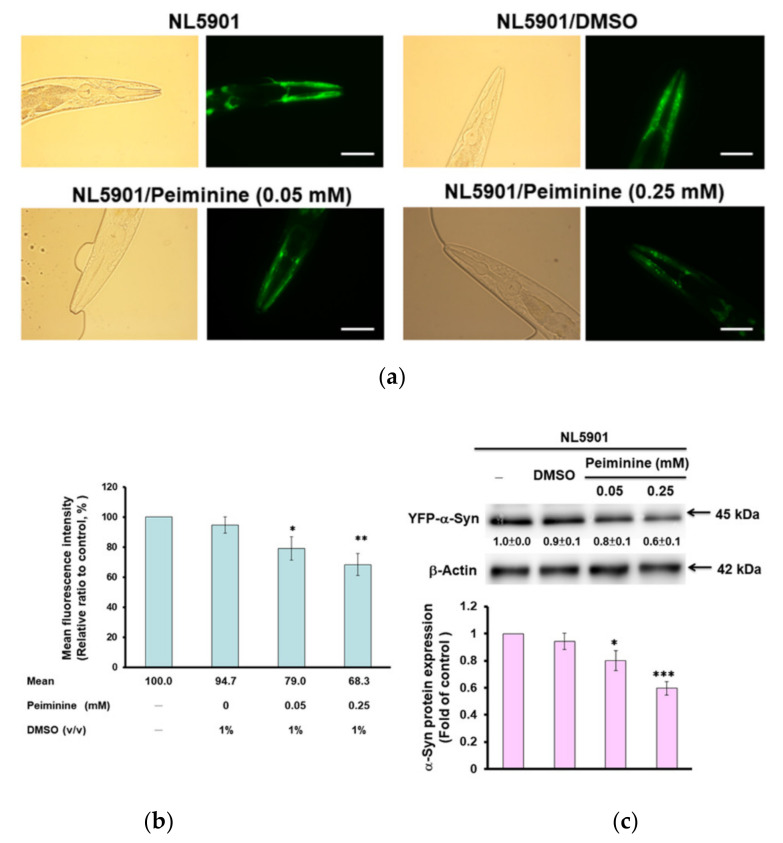
Accumulation of human α-synuclein in muscle cells of NL5901 worms was significantly reduced by treatment with peiminine (PMN). L1 stage NL5901 worms were cultured on OP50/NGM plates with or without PMN for 1 day. The worms were transferred to OP50/NGM/FUDR plates with or without PMN and cultured for 3 days, and then analyzed by fluorescence microscopy. (**a**) Representative YFP fluorescence images of the accumulation of α-synuclein in the head muscles of worms in each group. Scale bar = 50 µm. (**b**) ImageJ software was used to quantify the fluorescence intensity of YFP of each group (*n* = 50) in (**a**). (**c**) The protein levels of α-synuclein in each group were analyzed by Western blotting. The loaded internal control is the level of β-actin. The image shows representative data from one of three independent experiments. The relative ratio is expressed as the ratio of the level of α-synuclein in each group to that in the PMN-untreated group. * Indicates a significant difference between the PMN-treated group and the PMN-untreated group (* *p* < 0.05, ** *p* < 0.01, *** *p* < 0.001).

The aggregation and accumulation of human α-synuclein caused by overexpression or mutation of *SNCA* is a pathologic feature of PD. We used transgenic worms (NL5901) in which muscle cells express the YFP-fused protein of human α-synuclein for analysis. We found that PMN dose-dependently decreased the YFP fluorescence intensity of muscle cells of worm, which reflected the diminution in α-synuclein accumulation (Figure 4a). The fluorescence intensity of worms treated with 0.25 mM PMN was reduced by 33% compared with that in untreated worms (*p* < 0.01) (Figure 4b).

To clarify the reason for the reduced fluorescence intensity of α-synuclein-YFP in NL5901 worms treated with PMN, we performed Western blot analysis. The results indicate that PMN dose-dependently reduced the protein level of α-synuclein and therefore lessened its accumulation (Figure 4c), but did not directly interfere with its aggregation (data not shown). After treatment with 0.25 mM PMN, the level of α-synuclein in muscle cells was diminished by 38% in NL5901 worms compared with untreated worms (*p* < 0.001) (Figure 4c).

### 2.6. PMN Pretreatment Decreases the Level of Reactive Oxygen Species in 6-OHDA-Exposed N2 Worms and Enhances the Expression of Pink1 and Pdr-1

**Figure 5:**
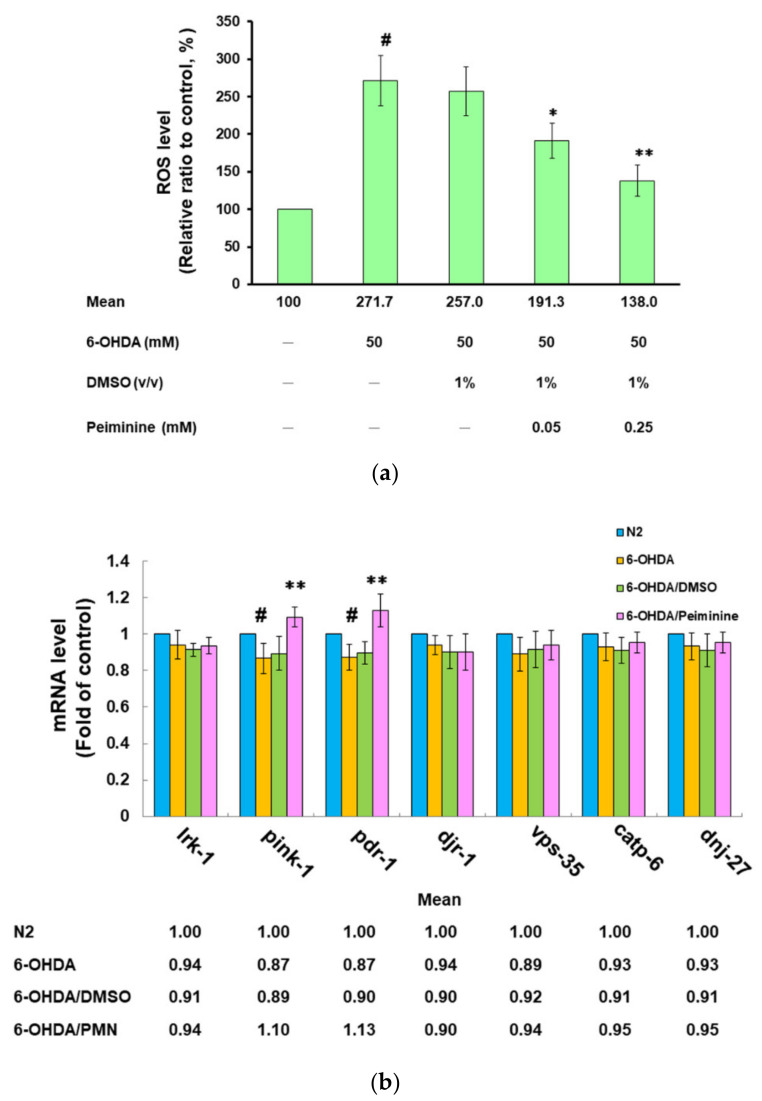
Pretreatment with peiminine (PMN) significantly lessened the level of reactive oxygen species (ROS) in 6-hydroxydopamine (6-OHDA)-exposed N2 worms and augmented the expression of *pink1* and *pdr-1*. L1 stage N2 worms were transferred to OP50/NGM plates with or without PMN and grown to the L3 stage, exposed to 6-OHDA for 1 h, and then transferred to OP50/NGM/FUDR plates with or without PMN and cultured for another 3 days for analysis. (**a**) Thirty worms randomly selected from each group were transferred to the wells of a 96-well plate, and the 2′,7′-dichlorodihydrofluorescein diacetate (H2DCFDA) probe was used to detect the level of intracellular ROS. ^#^ Indicates a significant difference between the 6-OHDA-exposed group and the control group (^#^ *p* < 0.001); ^*^Indicates a significant difference between the PMN- pretreated 6-OHDA-exposed group and the PMN-untreated 6-OHDA-exposed group (* *p* < 0.05, ** *p* < 0.01). (**b**) qPCR was used to quantify the mRNA levels of PD-related homologous genes in worms. ^#^Indicates a significant difference between the 6-OHDA-exposed group and the control group (^#^ *p* < 0.05); * Indicates a significant difference between the PMN-pretreated 6-OHDA-exposed group and the PMN-untreated 6-OHDA-exposed group (** *p* < 0.01).

Our previous studies showed that 6-OHDA exposure will increase the ROS in the worm, thus causing DA neuronal apoptosis and degeneration [30]. Therefore, we wanted to know whether PMN affected the ROS levels in 6-OHDA-exposed worms. Compared with that in the control group, the ROS level of 6-OHDA-exposed worms was significantly increased by about 2.7-fold (*p* < 0.001) (Figure 5a). PMN diminished the ROS levels of 6-OHDA-exposed worms in a dose-dependent manner. After pretreatment with 0.25 mM PMN, the ROS level was lessened by about 49.2% compared with that in the untreated group (*p* < 0.01) (Figure 5a).

In addition, we analyzed the expression of homologous genes in *C. elegans* that are known to be associated with the pathophysiology of human PD. Using real-time quantitative PCR (qPCR), the expression of *lrk-1*/*LRRK2, djr-1.1*/*djr-1.2*/*DJ-1, vps-35*/*VPS35, catp-6*/*ATP13A2*, and *dnj-27*/*DNAJC10*/*Hsp40* in the 6-OHDA-exposed group was not significantly different from that in the control group (Figure 5b). However, the level of *pink-1/PINK1* and *pdr-1*/*PREN*/*parkin* was slightly reduced (*p* < 0.05). In worms pretreated with 0.25 mM PMN, the mRNA levels of *pink-1* and *pdr-1* were augmented by 1.3-fold (*p* < 0.01), respectively, compared with the PMN-untreated 6-OHDA-exposed group (Figure 5b).

### 2.7. PMN Treatment Enhances Proteasome Activity, Autophagy, and expression of Pdr-1 in NL5901 Worms

**Figure 6:**
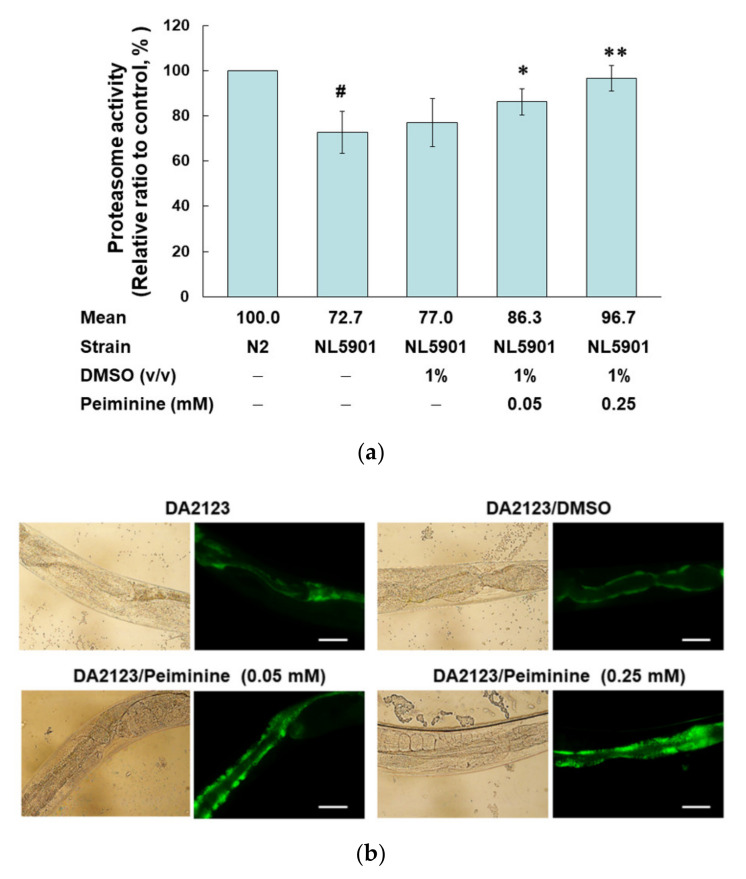

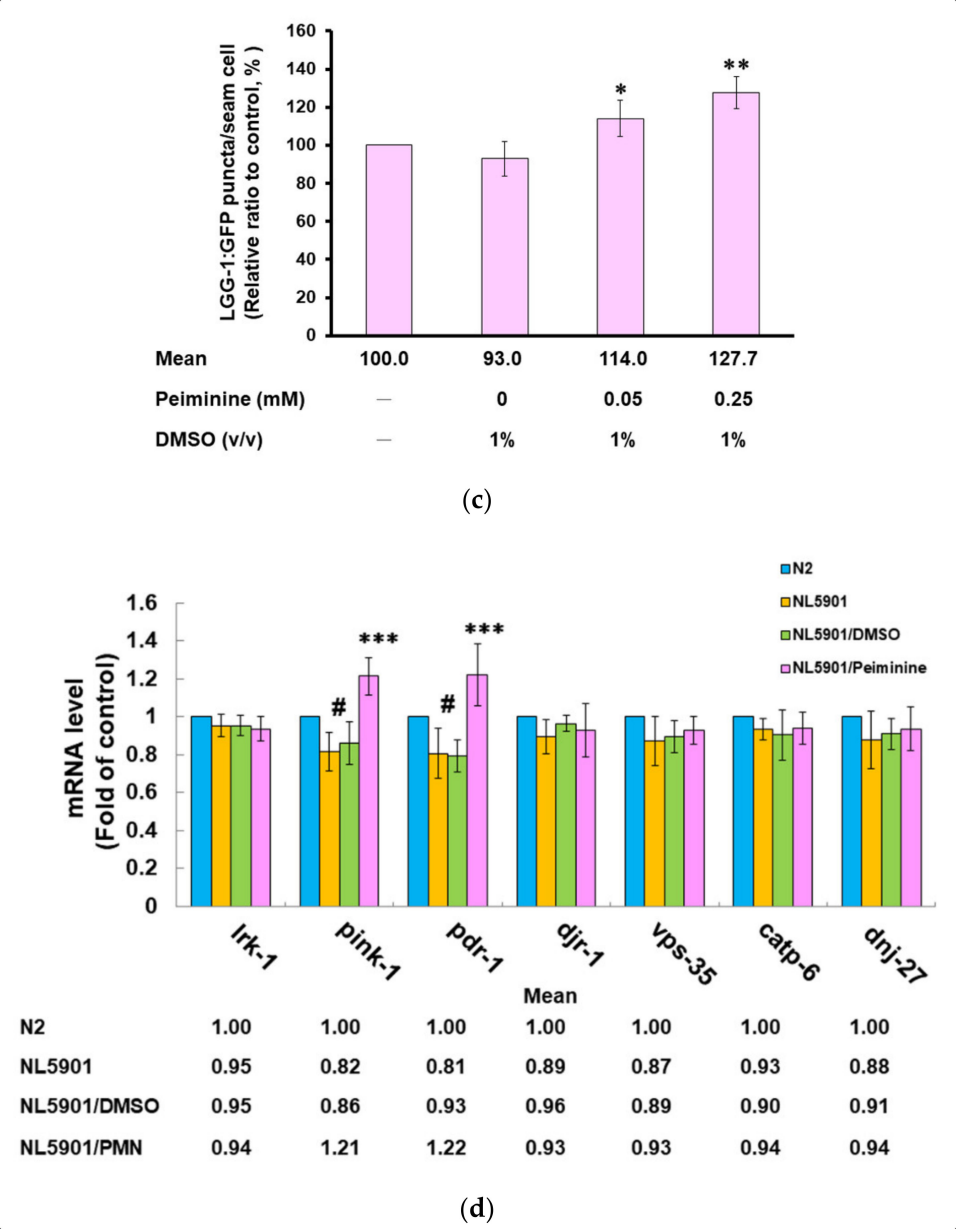
Treatment with peiminine (PMN) enhanced proteasome activity, autophagy, and *pdr-1* expression in NL5901 transgenic worms. (**a**) L1 stage NL5901 worms were cultured on OP50/NGM plates with or without PMN for 1 day. Worms were then transferred to OP50/NGM/FUDR plates with or without PMN and cultured for 3 days for analysis. The worm extracts of each group were tested for proteasome activity under the same amount of total protein. ^#^Indicates a significant difference between N2 and NL5901 worms (^#^ *p* < 0.01). * Indicates a significant difference between the PMN-treated group and the PMN-untreated group (* *p* < 0.05, ** *p* < 0.01). (**b**) L1 stage DA2123 worms were cultured on OP50/NGM plates with or without PMN for 1 day. Worms were then transferred to OP50/NGM/FUDR plates with or without PMN and cultured for 3 days for analysis. Representative images of the fluorescence distribution of positive puncta in the seam cells of each group of worm. Scale bar = 10 µm. (**c**) The number of positive puncta in the seam cells of DA2123 worms was calculated. At least 20 worms were calculated in each group, and at least 20 seam cells were calculated for each worm. * Indicates a significant difference between the PMN-treated group and the PMN-untreated group (* *p* < 0.05, ** *p* < 0.01). (**d**) The expression levels of PD-related genes in N2 and PMN-untreated or treated NL5901 worms were quantified by qPCR. ^#^Indicates a significant difference between N2 and NL5901 worms (^#^ *p* < 0.05). * Indicates a significant difference between the PMN-treated group and the untreated group (*** *p* < 0.001).

Previous studies have shown that the accumulation of α-synuclein can affect proteasome activity and autophagy, and that increasing the activity of the proteasome and autophagy can improve the cytotoxicity induced by the accumulation of α-synuclein [31]. Therefore, we used the fluorescent substrate of chymotrypsin to evaluate the effect of PMN on the proteasome activity of NL5901 worms. The basal level of proteasome activity in NL5901 worms was decreased by 27.3% compared with activity in N2 worms (*p* < 0.01) (Figure 6a). PMN treatment could enhance proteasome activity in a dose-dependent manner. Compared with the activity of the untreated group, treatment with 0.25 mM PMN increased the proteasome activity of worms 1.3-fold (*p* < 0.01) (Figure 6a).

In addition, we used transgenic DA2123 worms expressing LGG-1 (human LC3 homologous protein) fused with GFP derived by the *lgg-1* promoter to evaluate the autophagy activity by counting the number of fluorescent puncta formed in seam cells (Figure 6b). Compared with the number in untreated worms, the number of LGG-1::GFP puncta increased 1.28-fold (*p* < 0.01) after treatment with 0.25 mM PMN (Figure 6c).

Finally, we used qPCR to determine the expression levels of PD-related genes in N2 and PMN-untreated or treated NL5901 worms. Compared with N2 worms, except for the slightly lower expression of *pink-1* and *pdr-1* (*p* < 0.05), there was no significant difference in the basal level of expression of other genes in NL5901 worms (Figure 6d). After treatment with 0.25 mM PMN, the expression of *pink-1* and *pdr-1* in NL5901 worms was significantly increased by 1.5 fold (*p* < 0.001), respectively, compared with the untreated group (Figure 6d).

### 2.8. Inhibiting the Expression of Pdr-1 in Worms Can Reverse the Ability of PMN to Improve PD Pathology

**Figure 7:**
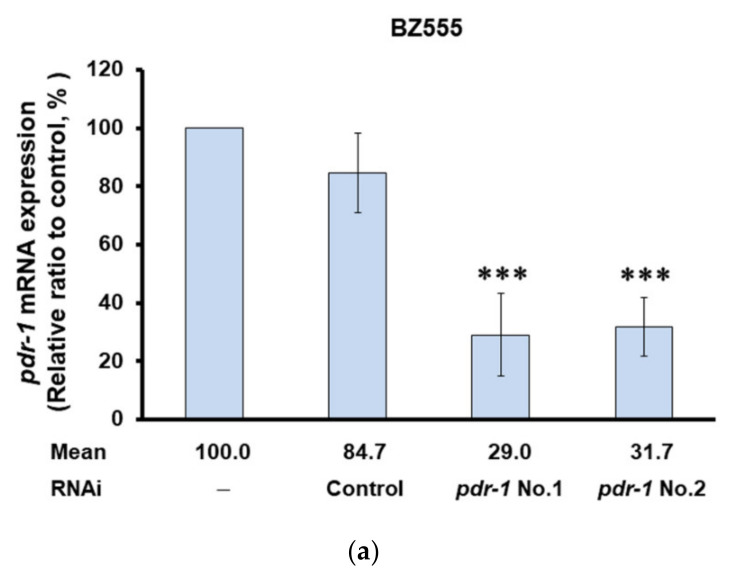

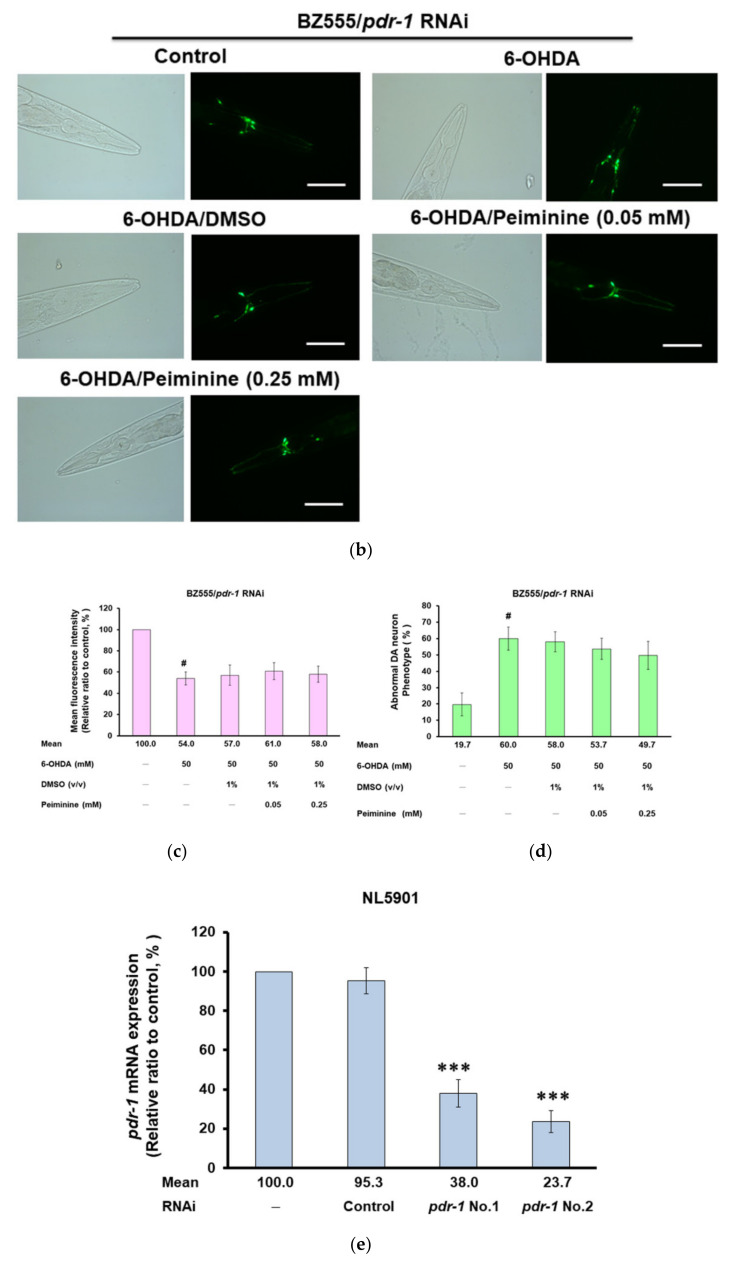

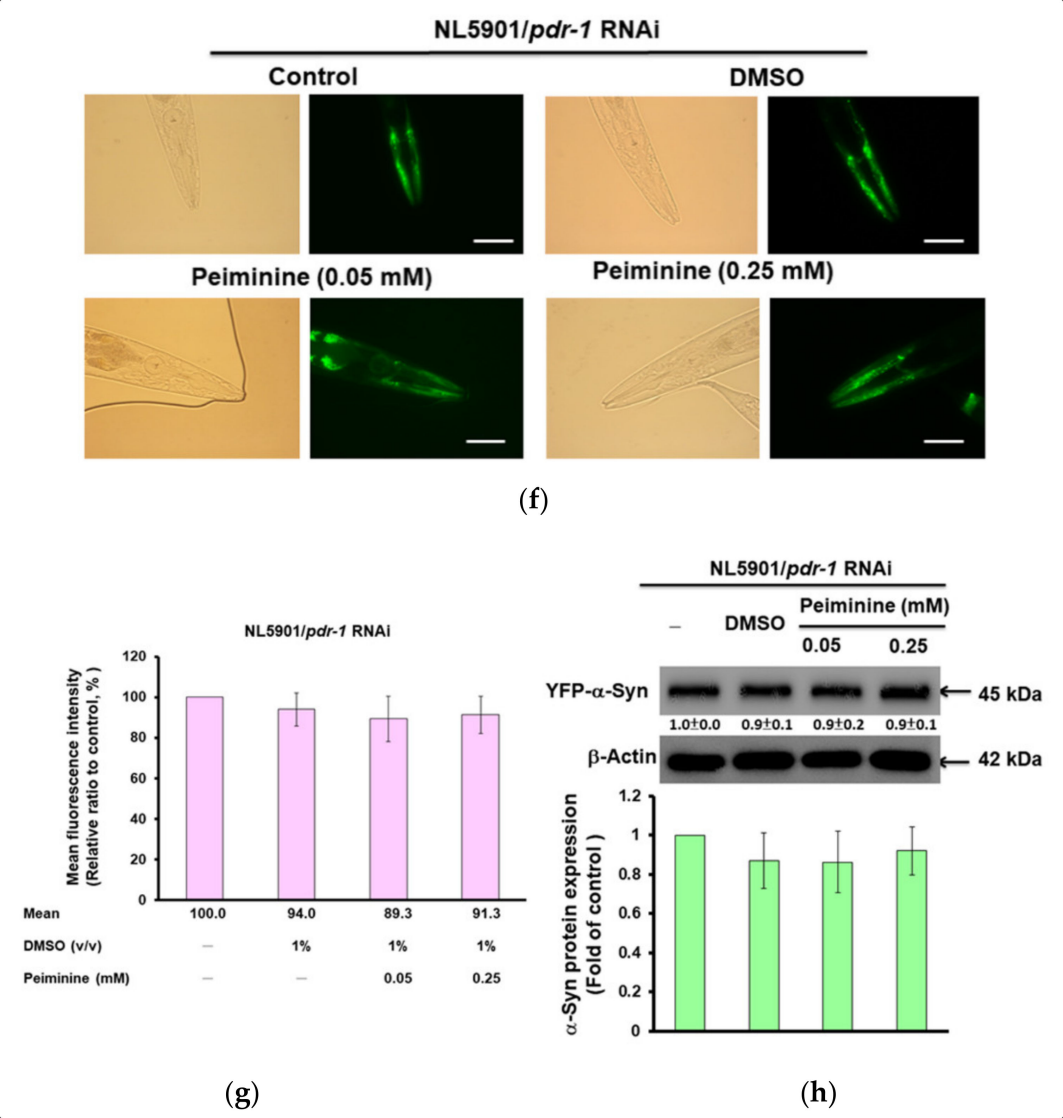
Using RNA interference (RNAi) to down-regulate the mRNA expression of *pdr-1* can abolish the ability of peiminine (PMN) to improve the pathology of Parkinson disease in worm models. In the 6-OHDA-exposed BZ555 model, L1 stage worms were transferred to *pdr-1* RNAi/NGM plates with or without PMN and grown to the L3 stage, exposed to 6-OHDA for 1 h, and then transferred to *pdr-1* RNAi/NGM/FUDR plates with or without PMN and cultured for another 3 days for analysis. In the transgenic NL5901 model, L1 stage worms were cultured on *pdr-1* RNAi/NGM plates with or without PMN for 1 day and then transferred to *pdr-1* RNAi/NGM/FUDR plates with or without PMN and cultured for 3 days. (**a**) The relative level of *pdr-1* mRNA in *pdr-1* RNAi-treated BZ555 worms was determined by qPCR. * Indicates a significant difference between the *prd-1* RNAi group and the control RNAi group (*** *p* < 0.001). (**b**) Representative fluorescence images of GFP expression patterns in DA neurons of BZ555 worms in each group. Scale bar = 50 µm. (**c**) ImageJ software was used to quantify the fluorescence intensity of the GFP image in (**b**) (*n* = 50). (d) The DA neuron degeneration phenotype defects of each group in (**b**) were scored. The data are represented as a percentage of the total population of worms with defective DA neuron phenotypes. ^#^ Indicates a significant difference between the 6-OHDA-exposed group and the control group (^#^ *p* < 0.001). (**e**) The relative level of *pdr-1* mRNA in *pdr-1* RNAi-treated NL5901 worms was determined by qPCR. * Indicates a significant difference between the *prd-1* RNAi group and the control RNAi group (*** *p* <0.001). (**f**) Representative YFP fluorescence images of the accumulation of α-synuclein in the head muscles of worms in each group. Scale bar = 50 µm. (**g**) ImageJ software was used to quantify the YFP fluorescence intensity of each group (*n* = 50) in (**f**). ^#^ Indicates a significant difference between the PMN-treated group and the PMN-untreated group (^#^ *p* < 0.001). (**h**) The protein levels of α-synuclein in each group were analyzed by Western blotting. The loaded internal control is the level of β-actin. The image shows representative data from one of three independent experiments. The relative ratio is represented as the ratio of the level of α-synuclein in each group to that in the PMN-untreated group.

The above experiments showed that PMN can promote the expression of *pdr-1* in worms. Furthermore, we wanted to verify the main mediating role of *pdr-1* by use of RNAi in PMN-pretreated 6-OHDA-exposed BZ555 worms and PMN-treated NL5901 worms. In BZ555 worms, the mRNA expression of *pdr-1* in the *pdr-1* RNAi group was reduced by 71% (No. 1, *p* < 0.001) compared to that in the control RNAi group (Figure 7a). After *pdr-1* down-regulation, GFP fluorescence intensity did not increase in PMN-pretreated 6-OHDA-exposed BZ555 worms compared with that in the PMN-untreated 6-OHDA-exposed group, reflecting that PMN lost the ability to improve DA neuron degeneration (Figure 7b–d).

In NL5901 worms, the expression of *pdr-1* in the *pdr-1* RNAi group was reduced by 76.3% (no. 2, *p* < 0.001) compared with expression in the RNAi control group (Figure 7e). After *pdr-1* down-regulation, YFP fluorescence intensity did not decline in PMN-treated NL5901 worms compared with that in the PMN-untreated group, reflecting that the accumulation of α-synuclein was not improved (Figure 7f,g). In Western blotting analysis, after *pdr-1* was down-regulated, PMN-treated NL5901 worms did not show a lessening in human α-synuclein protein levels compared with the PMN-untreated group (Figure 7h), reflecting that the amount of α-synuclein was not reduced.

### 2.9. PMN Treatment Improves the Toxicity of 6-OHDA Exposure and α-Synuclein Overexpression in the SH-SY5Y Cell Line

**Figure 8:**
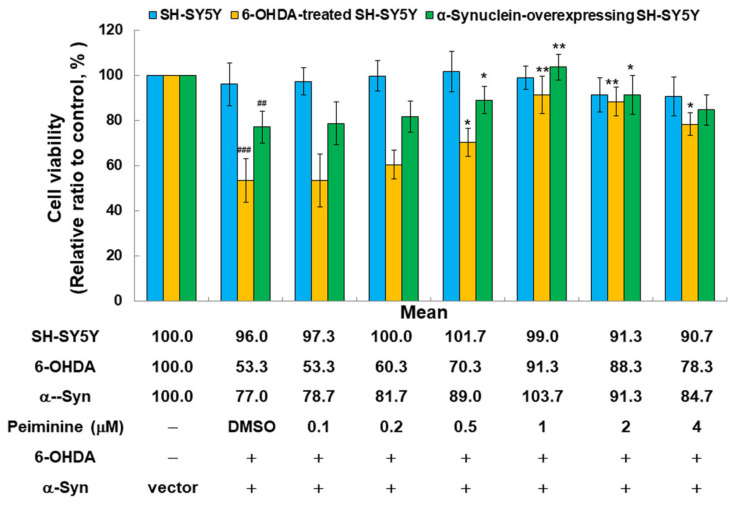
PMN treatment reduces the toxicity of 6-OHDA exposure and α-synuclein overexpression in the SH-SY5Y cell line. Cells were treated with 0.1, 0.2, 0.5, 1, 2, or 4 μM PMN for 24 h (SH-SY5Y group), followed by 6-OHDA exposure for 18 h (6-OHDA-treated SH-SY5Y group), or α-synuclein-overexpressing cells were treated with PMN for 24 h (α-synuclein-overexpressing SH-SY5Y group), and the cell survival rate was determined by MTT assay. ^#^ Indicates a significant difference between the 6-OHDA-exposed group or α–synuclein-overexpressing group and the control group (^##^ *p* < 0.01, ^###^ *p* < 0.001). * Indicates a significant difference between the PMN-treated group and the PMN-untreated group (* *p* < 0.05, ** *p* < 0.01).

To further evaluate the efficacy of PMN in improving PD, we used a human SH-SY5Y cell line with 6-OHDA exposure and α-synuclein overexpression. According to the results of the MTT assay, treatment with 4 μM PMN was not toxic to SH-SY5Y cells (Figure 8). In addition, the viability of 6-OHDA-exposed and α-synuclein-overexpressing cells was reduced by 47% (*p* < 0.001) and 23% (*p* < 0.01), respectively, compared with the control group (Figure 8). When the 6-OHDA-exposed and α-synuclein-overexpressing cells were treated with 1 μM PMN, their viability was increased 1.7-fold (*p* < 0.01) and 1.3-fold (*p* < 0.01), respectively, compared with the PMN-untreated groups (Figure 8).

### 2.10. Down-Regulation of Parkin Abolishes the Anti-Apoptotic ability of PMN in 6-OHDA-Exposed SH-SY5Y Cells

**Figure 9:**
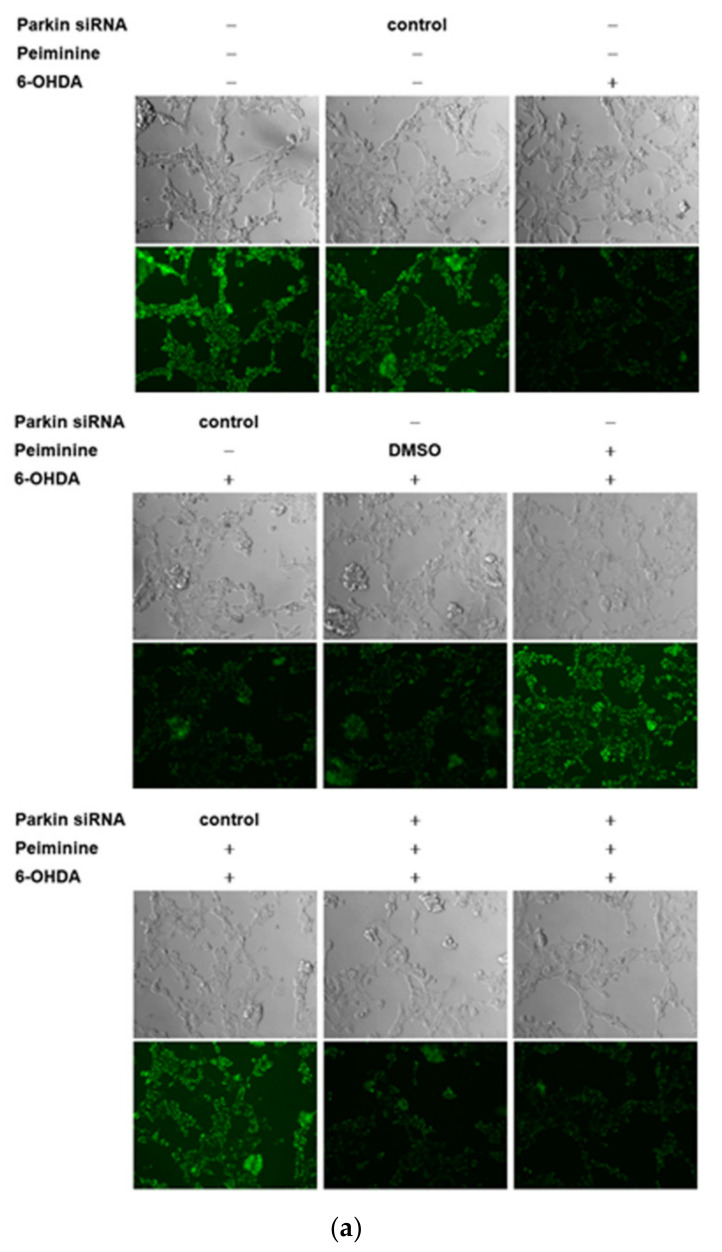

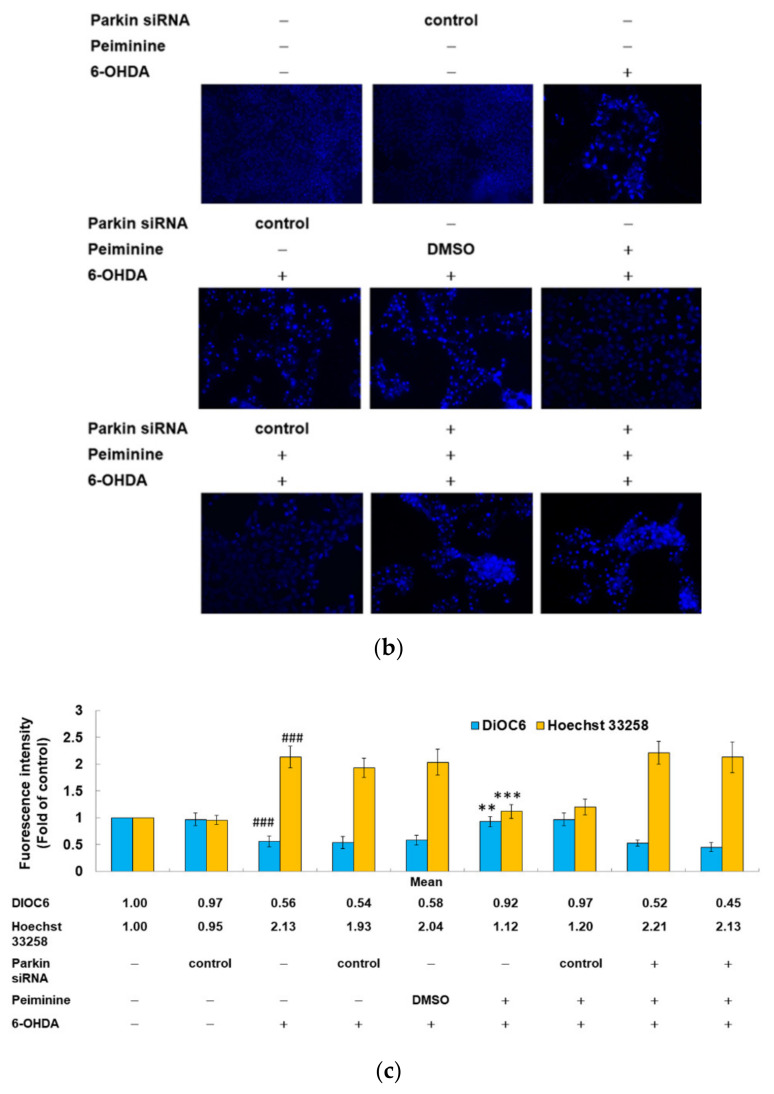

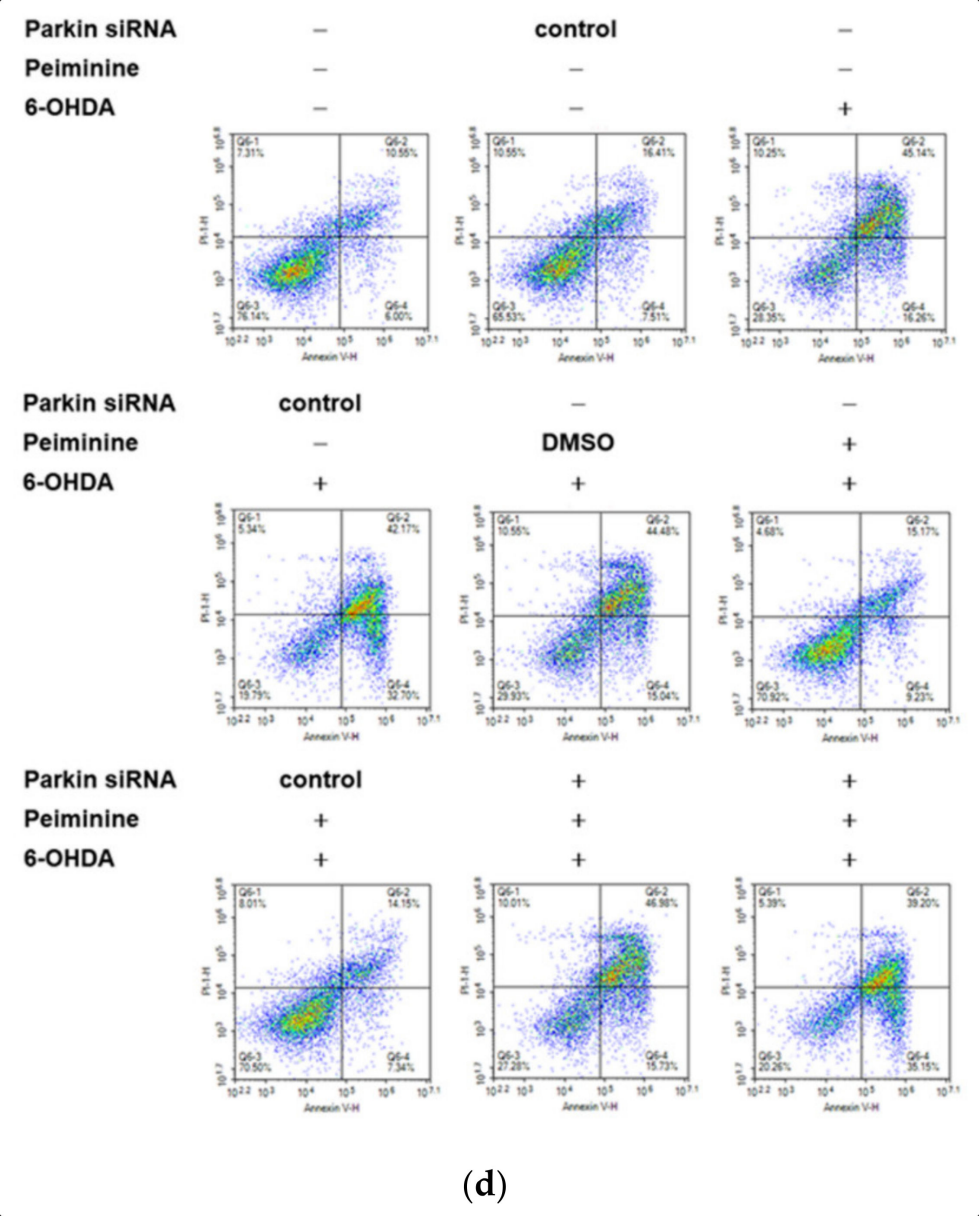
Down-regulation of *parkin* by RNAi can reverse the anti-apoptotic effect of peiminine (PMN) in 6-hydroxydopamine (6-OHDA)-exposed SH-SY5Y cells. SH-SY5Y cells were transfected with control siRNA or *parkin* siRNA for 24 h, and then the transfected cells were treated with 1 μM PMN or no treatment, respectively. After 24 h, cells were exposed to 100 μM 6-OHDA for 18 h. (**a**) DiOC6 (1 μM) staining was used to evaluate damage to mitochondrial membrane potential (MMP). The representative fluorescence images of each group are shown here. (**b**) Hoechst 33258 (5 μg/mL) staining was used to evaluate the ratio of nuclear condensation. The representative fluorescence images of each group are shown here. (**c**) ImageJ software was employed to analyze the fluorescence intensity of the staining in (**a**) and (**b**). The relative fluorescence intensity is indicated as a ratio relative to the control group. (**d**) Flow cytometric analysis of Annexin-V-FITC and PI staining was implemented on each group. Apoptosis rate = percentage of early apoptotic cells in the lower right quadrant + percentage of late apoptotic cells in the upper right quadrant. The results shown above are the standard errors of the average of three independent experiments. # Indicates a significant difference between the 6-OHDA-exposed group and the control group (^###^*p* < 0.001). * Indicates a significant difference between the PMN-pretreated 6-OHDA-exposed group and the PMN-untreated 6-OHDA-exposed group (** *p* < 0.01, *** *p* < 0.001).

**Figure 10:**
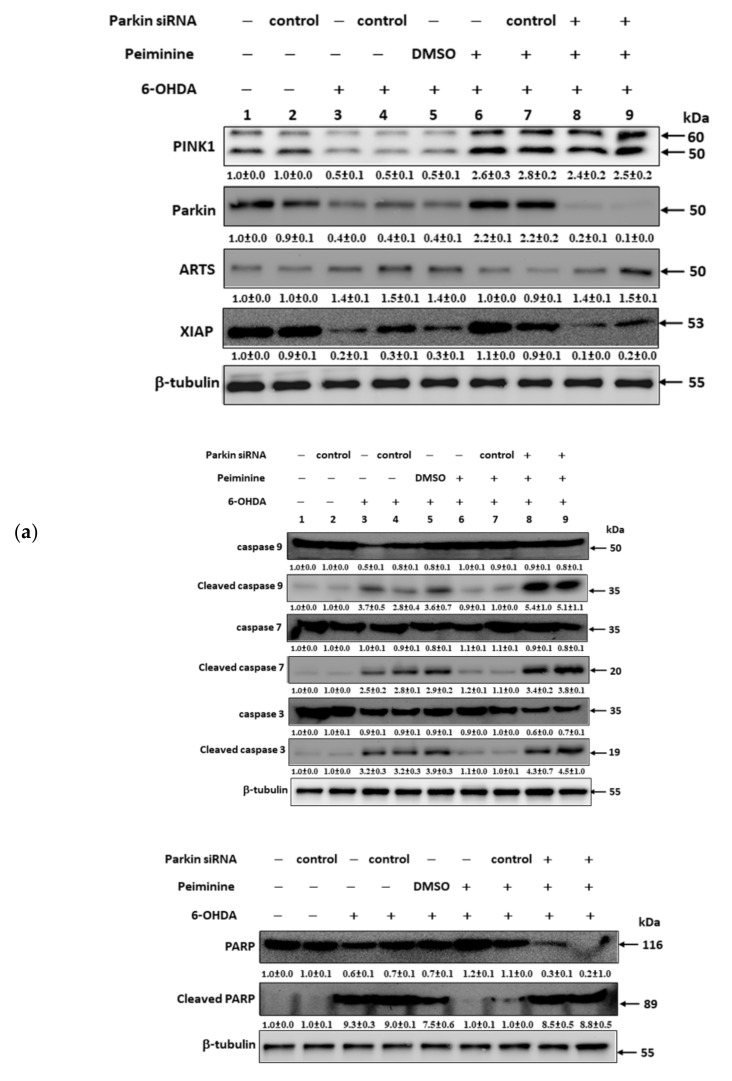

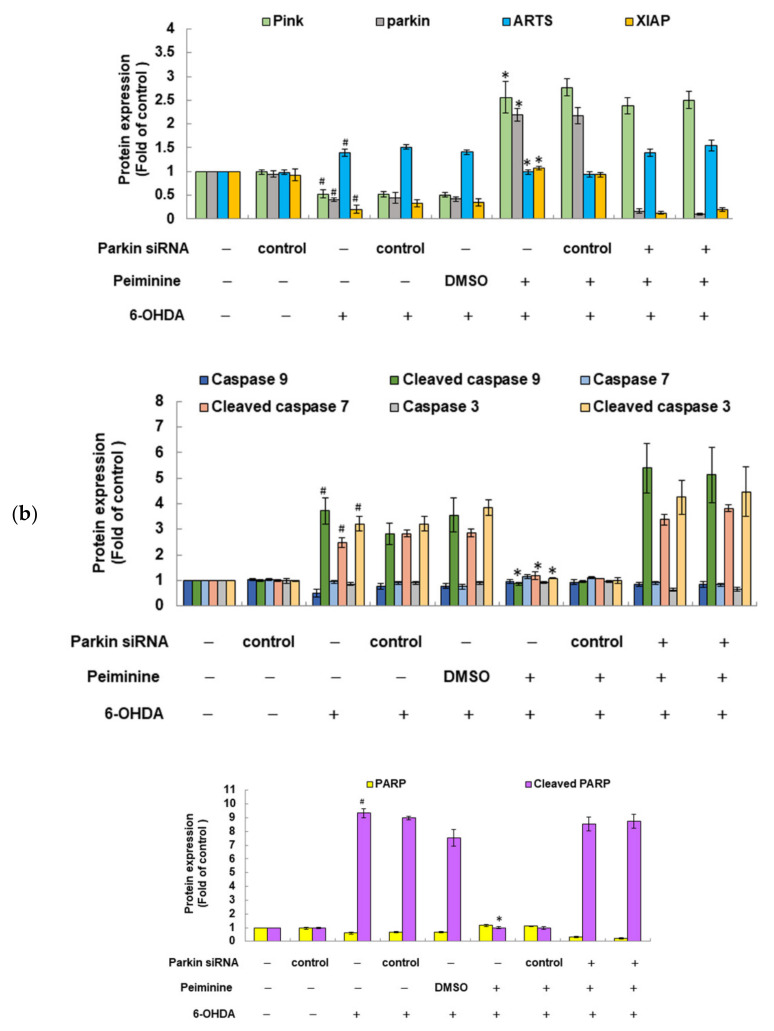
Western blot analysis was used to quantify the levels of PINK1, parkin, ARTS, XIAP, and apoptotic core proteins. SH-SY5Y cells were transfected with control siRNA or *parkin* siRNA for 24 h, and then the transfected cells were treated with 1 μM PMN or no treatment, respectively. After 24 h, cells were exposed to 100 μM 6-OHDA for 12 h. (**a**) Shows representative results from one of three independent experiments. (**b**) ImageJ software was utilized to quantify the signal intensity of the image in (**a**). The loaded internal control is the level of β-tubulin. The relative ratio is represented as the ratio of each group to the control group. ^#^ Indicates a significant difference between the 6-OHDA-exposed group and the control group (^#^ *p* < 0.001). * Indicates a significant difference between the PMN-pretreated 6-OHDA-exposed group and PMN-untreated 6-OHDA-exposed group (* *p* < 0.001).

The results of our research in the worm model confirmed that PMN can ameliorate ROS toxicity and DA neuron death caused by 6-OHDA exposure, which may inhibit cell apoptosis by inducing the expression of *pdr-1* (a human parkin homologous gene). We further used the SH-SY5Y cell model to verify this result. DiOC6 and Hoechst 33258 analysis showed that compared with the unexposed group, the 6-OHDA-exposed group had a 44% reduction in mitochondrial membrane potential (MMP, *p* < 0.001) (Figure 9a,c) and 2.1-fold (*p* < 0.001) greater nuclear condensation (Figure 9b,c). To quantify apoptosis, we used Annexin-V-FITC/PI flow cytometry analysis. As shown in Figure 9d, compared with that in the control group, apoptosis was 3.7-fold greater in the 6-OHDA-exposed group (*p* < 0.001). Western blot analysis showed that compared with levels in the control group, the protein levels of PINK1 and parkin were decreased by 47.0% (*p* < 0.001) and 59.7% (*p* < 0.001), respectively, in the 6-OHDA-exposed group. The levels of cleaved caspase 9, caspase 7, caspase 3, and PARP associated with apoptosis were 3.7-fold (*p* < 0.001), 2.5-fold (*p* < 0.001), 3.2-fold (*p* < 0.001), and 9.3-fold (*p* < 0.001) greater, respectively (Figure 10a,b).

However, PMN pretreatment significantly lessened the apoptosis of SH-SY5Y cells induced by 6-OHDA. Compared with that of PMN-untreated 6-OHDA-exposed cells, the MMP of 1 μM PMN-treated 6-OHDA-exposed cells was 1.7-fold greater (*p* < 0.01) (Figure 9a,c). Nuclear condensation was reduced by 47% (*p* < 0.001) (Figure 9b,c), and apoptosis was significantly diminished by 60% (*p* < 0.001) (Figure 9d). In addition, the protein levels of PINK1 and parkin were augmented 4.8-fold (*p* < 0.001) and 5.5-fold (*p* < 0.001), respectively. The levels of cleaved caspase 9, caspase 7, caspase 3, and PARP were reduced by 76% (*p* < 0.001), 52% (*p* < 0.001), 66% (*p* < 0.001), and 89% (*p* < 0.001), respectively (Figure 10a,b). Finally, we used RNAi to inhibit the expression of *parkin* in SH-SY5Y cells. We found that the ability of PMN to reverse 6-OHDA-induced apoptosis was blocked (Figure 9 and Figure 10).

### 2.11. Down-Regulation of Parkin Can Reverse the Ability of PMN to Enhance Ubiquitin-Proteasome System Activity and Autophagy in α-Synuclein-Overexpressing SH-SY5Y Cells

**Figure 11:**
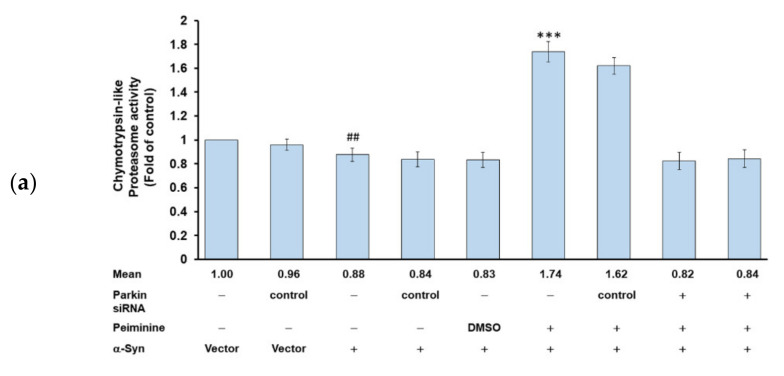

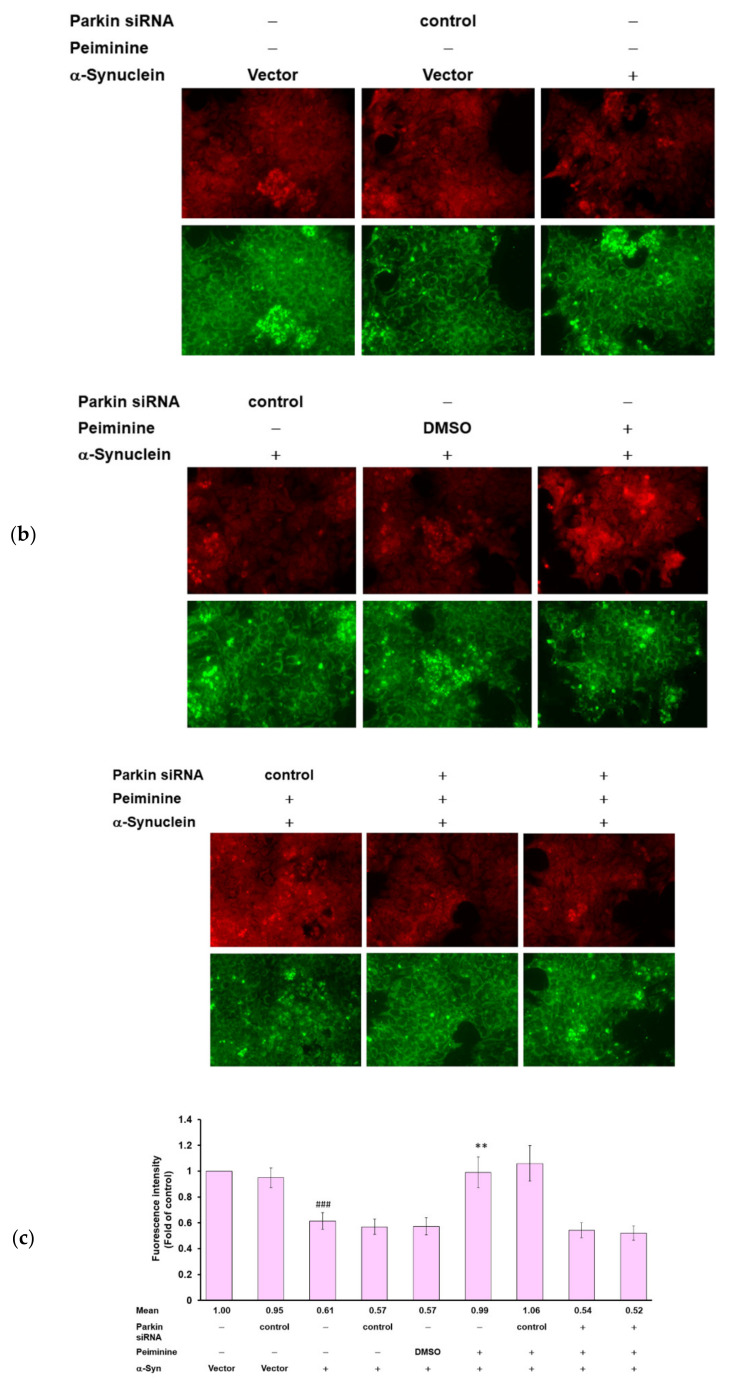

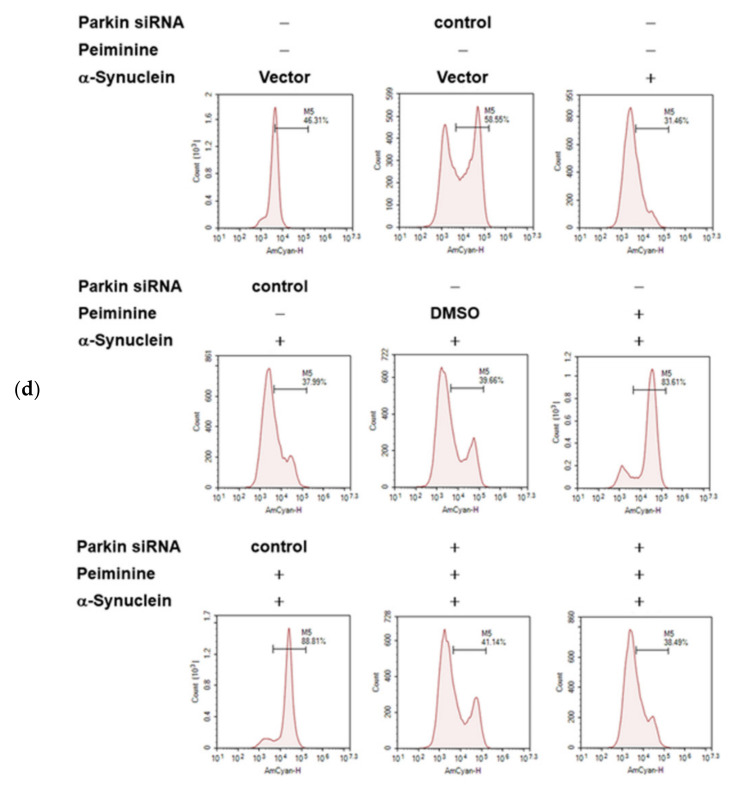
Down-regulation of parkin can abolish the ability of PMN to promote UPS activity and autophagy in α-synuclein-overexpressing SH-SY5Y cells. α-Synuclein-overexpressing SH-SY5Y cells were transfected with control siRNA or *parkin* siRNA for 24 h. Next, the transfected cells were treated with 1 μM PMN or untreated for 24 h and then analyzed. (**a**) Suc-Leu-Leu-Val-Tyr-AMC was used as a substrate for measuring proteasome activity. The relative fold of the proteasome activity is represented as the ratio of the activity of each group to the control group. (**b**) Acridine orange staining was used to evaluate the number of acidic vesicular organelles in cells. The representative fluorescence images of each group are shown here. (**c**) The fluorescence intensity of the staining in (**b**) was analyzed by employing ImageJ software. The relative fluorescence intensity is shown as the ratio of the fluorescence intensity of each group to that of the control group. (**d**) Cells were incubated with the Autophagosome Detection Reagent for 30 min and analyzed by flow cytometry. The results shown above are the standard errors of the average of three independent experiments. ^#^Indicates a significant difference between the α-synuclein-overexpressing group and the control group (^##^ *p* < 0.01, ^###^ *p* < 0.001). * Indicates a significant difference between the PMN-pretreated group and the PMN-untreated group (** *p* < 0.01, *** *p* < 0.001).

**Figure 12:**
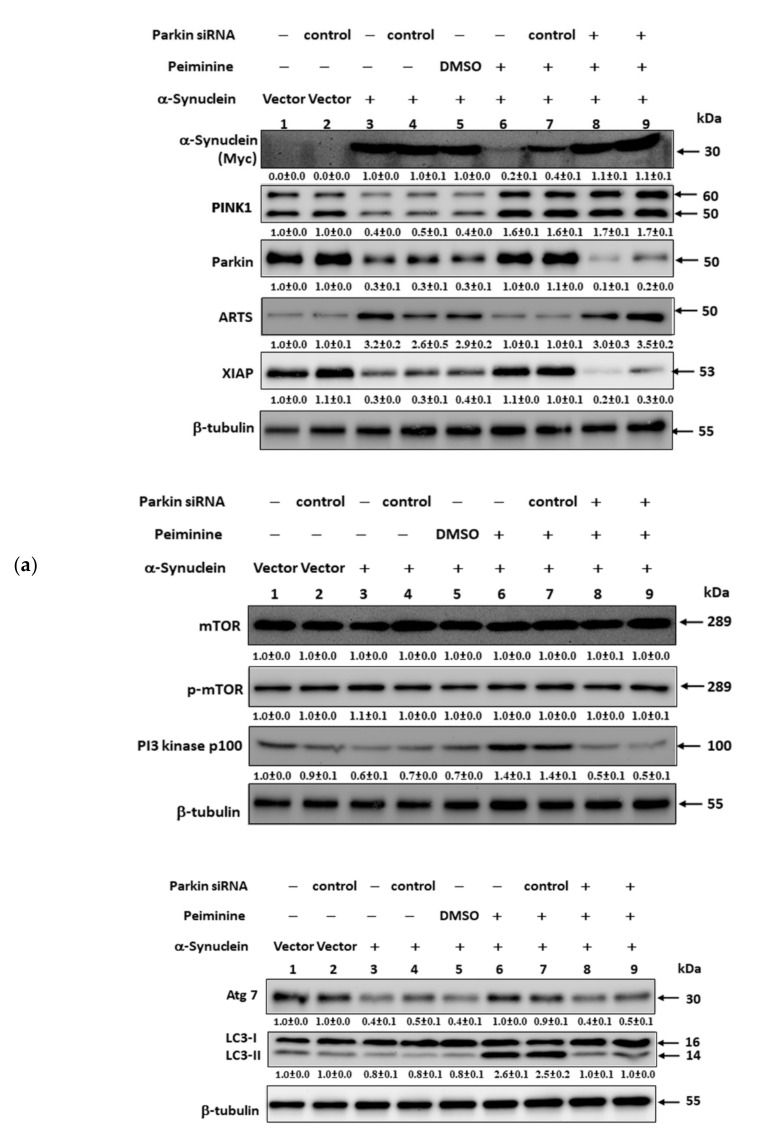

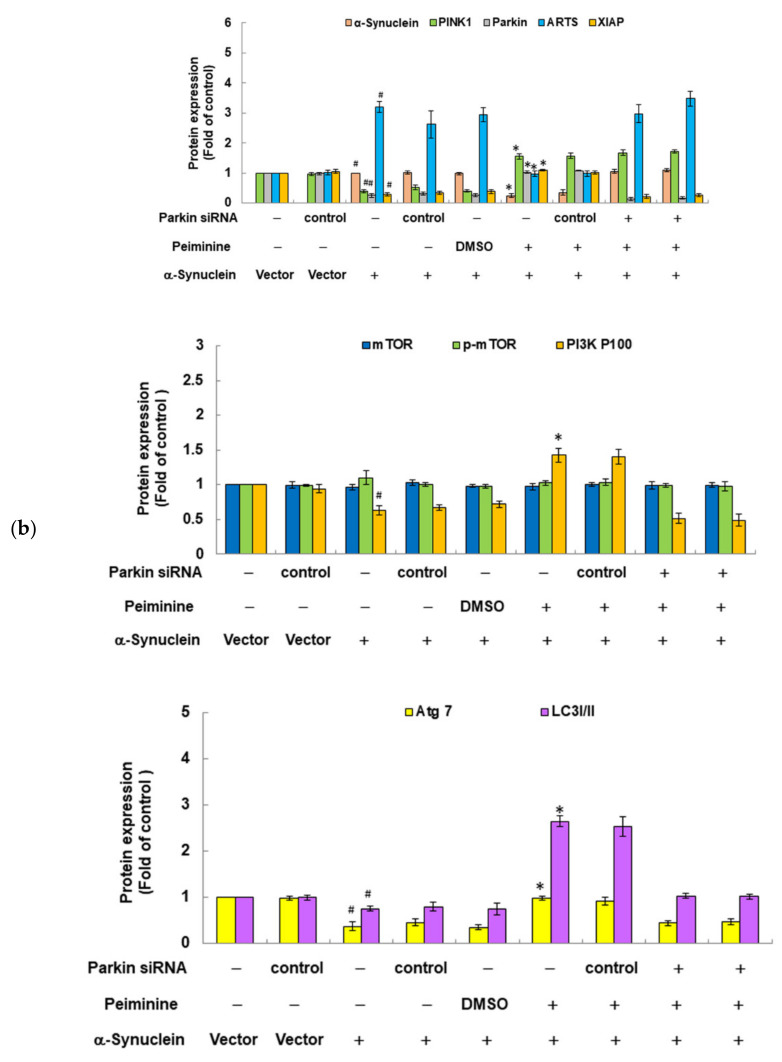
Western blot analysis was used to quantify the protein levels of PINK1, parkin, ARTS, XIAP, and autophagy core proteins. α-Synuclein-overexpressing SH-SY5Y cells were transfected with control siRNA or parkin siRNA for 24 h. Next, the transfected cells were treated with 1 μM PMN or untreated for 24 h and then analyzed. (**a**) Shows representative results from one of three independent experiments. (**b**) ImageJ software was utilized to quantify the signal intensity of the image in (**a**). The loaded internal control was the level of β-tubulin. The relative ratio is represented as the ratio of each group to the control group. ^#^ Indicates a significant difference between the α-synuclein-overexpressing group and the control group (^#^ *p* < 0.001). * Indicates a significant difference between the PMN-treated group and the PMN-untreated group (* *p* < 0.001).

Since we confirmed in the above experiments that PMN can improve the accumulation of α-synuclein in NL5901 worms, we hypothesized that it may be associated with inducing the expression of *pdr-1* to enhance the UPS and the activity of autophagy. We further used the human α-synuclein-overexpressing SH-SY5Y cell model to verify this result. First, we constructed the pcDNA 3.1-SNCA-Myc plasmid and transfected it into the SH-SY5Y cell line to obtain an α-synuclein-overexpressing cell model [31]. Compared with values in the control group, in α-synuclein-overexpressing cells, the UPS activity and the fluorescence intensity of acidic vesicle organelle staining were decreased by 12% (*p* < 0.01) (Figure 11a) and 39% (*p* < 0.001) (Figure 11b,c), respectively. To quantify the autotrophic defect caused by overexpression of α-synuclein, we used LC3II-based flow cytometry. As shown in Figure 11d, compared with the control group, overexpression of α-synuclein caused a 32% reduction in the autophilic activity of SH-SY5Y cells (*p* < 0.01). Western blot analysis showed that compared with the control group, PINK1 and parkin were reduced by 60% (*p* < 0.001) and 73% (*p* < 0.001) in α-synuclein-overexpressing cells, respectively (Figure 12a,b). The autophagy-related proteins PI3K p100, Atg7, and LC3I/LC3II were lessened by 37% (*p* < 0.001), 62% (*p* < 0.001), and 25% (*p* < 0.001), respectively. There were no significant changes in mTOR or p-mTOR (Figure 12a,b).

PMN treatment improved the defects in the UPS and autophagy caused by α-synuclein overexpression in SH-SY5Y cells. The results show that compared with the PMN-untreated group, the UPS activity, the fluorescence intensity of acidic vesicular organelle staining, and autophilic activity increased by 2.0-fold (*p* < 0.001) (Figure 11a), 1.6-fold (*p* < 0.01) (Figure 11b,c), and 2.7- fold (*p* < 0.001) (Figure 11d) in the PMN-treated group, respectively. Western blot analysis indicated that in the PMN-treated group compared with the untreated group, the protein level of PINK1 and parkin increased by 2.9-fold (*p* < 0.001) and 3.8-fold (*p* < 0.001), respectively (Figure 12a,b). The expression of PI3K p100, Atg7, and LC3I/LC3II was augmented by 2.3-fold (*p* < 0.001), 2.6-fold (*p* < 0.001), and 3.5-fold (*p* < 0.001), respectively (Figure 12a,b). There was no significant change in mTOR or p-mTOR (Figure 12a,b). Finally, we used RNAi to down-regulate the expression of *parkin* in α-synuclein-overexpressing cells. The results revealed that the ability of PMN to activate the UPS and autophagy was inhibited (Figure 11 and Figure 12).

### 2.12. PMN May Contribute to Anti-Parkinson Activity by Up-Regulating Parkin Performance, Leading to a Diminution of Apoptosis-Related Protein in the TGF-β Signaling Pathway (ARTS) and a Rise in X-Linked Inhibitor of Apoptosis (XIAP)

In worm and cell models with 6-OHDA exposure and α-synuclein overexpression, we confirmed that PMN can enhance parkin expression. Parkin is known as an E3 ubiquitin ligase, which can regulate the activity of downstream substrates through selective ubiquitination. Through extensive analysis, we found that one of its substrates, ARTS, may also be involved in the neuroprotective mechanism of PMN. Using Western blot analysis, we showed that ARTS protein expression in 6-OHDA-exposed cells was 1.4-fold (*p* < 0.001) that of control cells (Figure 10). In PMN-treated 6-OHDA-exposed cells, ARTS protein expression was reduced by 29.0% (*p* < 0.001) compared with PMN-untreated 6-OHDA-exposed cells (Figure 10). Using RNAi to down-regulate the expression of parkin in SH-SY5Y cells, we found that the ability of PMN to reverse the 6-OHDA-induced increase in ARTS expression was abolished (Figure 10). In α-synuclein-overexpressing cells, ARTS protein expression increased by 3.2-fold (*p* < 0.001) compared with that in control cells (Figure 12). In α-synuclein-overexpressing cells, the expression of ARTS protein was reduced by 69.0% (*p* < 0.001) in the PMN-treated group compared with the untreated group (Figure 12). RNAi was used to down-regulate the expression of *parkin* in α-synuclein-overexpressing SH-SY5Y cells. We found that the ability of PMN to reverse the increase in ARTS induced by overexpression of α-synuclein was abolished (Figure 12).

Several reports have shown that the function of XIAP, which can regulate apoptosis and promote proteasome activity and autophagy, is inhibited by binding of ARTS [15,16,17]. We wanted to further analyze whether PMN could affect the level of XIAP. Western blot analysis showed that XIAP protein expression in 6-OHDA-exposed cells was lessened by 79.3% (*p* < 0.001) compared with that in control cells (Figure 10). In PMN-treated 6-OHDA-exposed cells, XIAP protein expression was 5.2-fold (*p* < 0.001) that in PMN-untreated 6-OHDA-exposed cells (Figure 10). Using RNAi to down-regulate the parkin expression of cells, we found that the ability of PMN to reverse 6-OHDA-induced XIAP reduction was abolished (Figure 10). In α-synuclein-overexpressing cells, XIAP protein expression was reduced by 70.3% compared with that in control cells (*p* < 0.001) (Figure 12). In α-synuclein-overexpressing cells, XIAP protein expression in the PMN-treated group was 3.7-fold (*p* < 0.001) that in the untreated group (Figure 12). Using RNAi to down-regulate the *parkin* expression of cells, we found that the ability of PMN to reverse the diminished expression of XIAP induced by overexpression of α-synuclein was abolished (Figure 12).

## 3. Discussion

Previous studies have shown that PMN has rich biopharmaceutical activities, which can lead to resistance to oxidation, inhibition of inflammation, and blocking of tumor growth [32,33]. Here, we confirmed another activity of PMN: namely, a neuroprotective effect. PMN can inhibit 6-OHDA-induced ROS production, the decline in MMP, and cell apoptosis both in vivo and in vitro, thus preventing DA neuron degeneration and improving DA-related food-sensing behavior and lifespan defects in a worm model. We also showed that PMN can enhance the UPS and autophagy activity, thus reducing the accumulation of α-synuclein. This is the first time that PMN has been shown to have PD pathology-inhibiting features in animal and cell models. We also explored the possible molecular mechanisms of PMN through RNAi experiments. PMN may activate the PINK1 (worm homologous gene *pink1*)/parkin (worm homologous gene *pdr-1*) pathway and regulate the activity of their downstream substrates, such as enhancing the ubiquitination and degradation of ARTS (worm homologous genes *unc-59* and *unc-61*). This in turn increases the level of XIAP (worm homologous gene *iap*), preventing 6-OHDA-induced apoptosis and excessive accumulation of α-synuclein.

Research has shown that *PINK1* deficiency impairs the differentiation of DA neurons from adult neural stem cells [34] and inhibits the mitophagy and MMP of DA neurons [35]. Knockout of *PINK1* affects the neurotransmission of DA neurons and causes motor dysfunction in *Drosophila* [36]. In *PINK1* knockout mice, the aggregation of abnormal endogenous α-synuclein is increased, which increases the sensitivity of DA neurons to α-synuclein and finally causes degeneration [37]. In addition, *PINK1* knockout mice have less tyrosine hydroxylase in the hippocampus, which changes DA signaling in the hippocampus and causes damage to learning and memory [38]. In aging *PINK1* knockout mice, mitochondrial DNA heteroplasmy in the substantia nigra is increased [39]. Other studies have shown that loss of PINK1 and overexpression of α-synuclein can lead to a diminution in the length of neurites in midbrain neurons, which may be related to mitochondrial fission and increased Golgi fragmentation [40]. Drp1 is a substrate of PINK1, and PINK1-mediated phosphorylation of Drp1^S616^ can directly increase mitochondrial fission, independent of parkin and autophagy. Some PINK1-related cases of familial and sporadic PD have been shown to have decreased phosphorylation of Drp1^S616^ [41]. Therefore, PMN can enhance the neuroprotective effect by increasing the expression of PINK1.

Aging *parkin* knockout mice display dyskinesias, including hindlimb defects and neuronal loss. In the DA neurons of these mice, the internal structure of the mitochondria is abnormal and fragmented [42]. Moreover, loss of parkin function results in an intrastriatal reconfiguration of interneuronal circuits and amplifications of synchronized cortico-striatal oscillations. These changes predispose the animal to an imbalance in striatal outflow [43]. FBP1 and AIMP are substrates of parkin, and both have been found to accumulate in the brains of PD patients. Their function is to transcriptionally activate the deubiquitinase USP29. The substrate for USP29 is myb binding protein 1A (MYBBP1A). In SH-SY5Y cells, knockout of parkin increases the level of AIMP2, leading to the accumulation of USP29 and MYBBP1A, which may be one reason for the pathogenesis of PD [44]. Studies have shown that the expression of parkin in neuronal cells is down-regulated by exogenous α-synuclein, which causes mitochondrial dysfunction [45] and neuroinflammation [46]. Interestingly, oxidative stress induces the post-translational modification of the cysteine of parkin in the substantia nigra of mice and humans, causing it to oxidize with age and gradually become insoluble. This oxidative modification is associated with neuroprotection, including reducing H_2_O_2_, neutralizing reactive DA metabolites, chelating free radicals in insoluble aggregates, and increasing melanin formation. Therefore, *parkin* mutants may lose the ability to complement this redox effect, which increases oxidative stress and causes DA neurons to age, thereby increasing the risk of PD [47]. Therefore, PMN can prevent neuronal damage and degeneration by improving expression of parkin.

Studies have reported iron deposits in the substantia nigra pars compacta of PD animal models and patients. Thus, the destruction of iron homeostasis may be related to PD. Iron is reported to inhibit the activity of parkin, thereby hindering the degradation of α-synuclein by the proteasome. The resulting aggregated α-synuclein leads to mitochondrial dysfunction and apoptosis in SH-SY5Y cells [48]. Lactoferrin is a transferrin that regulates iron homeostasis. Parkin can bind to lactoferrin and degrade it by ubiquitination to affect iron homeostasis. Elevated levels of lactoferrin and its receptor have been observed in parkin mutant PD [49]. Divalent metal transporter 1 (DMT1) is also a substrate for parkin ubiquitination. α-Synuclein activates p38 mitogen-activated protein kinase (MAPK) to phosphorylate parkin ^S131^, thereby reducing the E3 ubiquitin ligase activity of parkin, leading to high DMT1 levels and abnormal iron accumulation [50]. Some PD patients show S-nitrosylated parkin (SNO-parkin). Studies in SH-SY5Y cells have shown that SNO-parkin lessens the ubiquitination activity for DMT1, leading to high levels of DMT1, thus causing abnormal iron accumulation and neurodegeneration [51]. Interestingly, reducing DMT-1 and iron accumulation may result in augmented iron regulatory protein (IRP)/iron responsive element (IRE) interactions on the 5’UTR of *SNCA* to lower its translation. Therefore, the abnormal increase in iron in the brain of PD patients promotes the development of iron attenuating agents and iron chelators as a new treatment strategy for PD [52]. PMN can upregulate the expression of parkin and may maintain the stable concentration of iron ions in nerve cells and prevent iron and as well as α-synuclein accumulation from causing damage to the cells. In the future, we will further use DA neurons derived from iPS stem cell of PD patient which over express endogenous SNCA, to evaluate the efficacy of PMN against α-synucleinopathies.

DA neurons derived from induced pluripotent stem cell lines from patients with *parkin* mutations have mitophagy and autophagy–lysosomal pathway defects [53,54], and the complexity and maturity of neurites is decreased by *parkin* mutations [55]. In the *Drosophila* model, PINK1 or parkin dysfunction can cause PARIS-dependent inhibition of PGC-1α and its downstream transcription factors NRF1 and TFAM in DA neurons, thereby blocking mitochondrial biogenesis [56]. It is worth noting that VDAC1 is also a substrate of parkin, which can regulate mitophagy and apoptosis. VDAC1 lacking monoubiquitination (K274R) promotes apoptosis by increasing mitochondrial calcium uniporter channel to promote calcium uptake. VDAC1 lacking polyubiquitination (Poly-KR) hinders mitophagy [57]. Recent studies in mouse models and humans have revealed that lack of parkin and PINK1 can cause mitochondrial damage, the release of mitochondrial DNA (mtDNA), and an increase in IL-6 expression, leading to inflammation [58]. In addition, the endoplasmic reticulum-mitochondrial contact site is a key structure for cell function. The site is involved in a large number of cellular processes, including Ca^2+^ signaling and selective degradation of mitochondria. PINK1/parkin is known to be involved in the mediation of this pathway [59]. Therefore, PMN can maintain the function and biogenesis of mitochondria in neuron by increasing the activity of parkin, avoiding its damage and aging.

In addition to our present findings on PMN, other studies have shown that various phytocompounds and plant proteins can overcome defects in PINK1 or parkin or regulate the expression of these genes for neuroprotection. For example, naringenin can improve the expression of PINK1 to diminish cellular oxidative stress and restore MMP, thereby alleviating 6-OHDA-induced toxicity in SH-SY5Y cells and zebrafish models [60]. In an MPTP-induced PD model, ursodeoxycholic acid modulates the PINK1/parkin pathway to improve mitochondrial function, inhibit apoptosis, and enhance autophagy, thus protecting DA neurons against oxidative stress [61]. Celastrol activates mitophagy by enhancing the performance of PINK1, thus preventing DA neuron death [62]. Ginseng protein prevents mitochondrial dysfunction and neurodegeneration by inducing mitochondrial unfolded protein response (UPR^mt^) in the PINK1-deficient *Drosophila* PD model [63]. Andrographolide can induce parkin-mediated mitophagy to inhibit the activation of NLRP3 inflammasomes in LPS-MPTP mice and microglia and thereby reduce the death of DA neurons [64]. Vasicinone can induce autophagy by enhancing the PINK/parkin pathway to prevent paraquat-induced mitochondrial dysfunction of DA neurons and reduce α-synuclein levels [65].

Our previous report indicated that substrates of parkin are involved in many important cellular physiologic processes, including apoptosis, mitochondrial metabolism, and protein clearance [66]. In this study, we found that the protein expression of ARTS, one of the substrates of parkin, is down-regulated by PMN. By contrast, the protein expression of XAIP, which is inhibited by ARTS binding, is up-regulated by PMN. Both proteins may be related to the mediation of parkin.

ARTS is related to the enhancement of apoptosis induced by TGF-β. When apoptosis occurs, ARTS is located in the mitochondria and is transported to the nucleus. The P loop mutation of ARTS can abolish its ability to activate caspase 3 and induce apoptosis [67]. ARTS is also a polymerizing GTP-binding protein, which can serve as a molecular scaffold. In Lewy bodies, ARTS co-localizes with α-synuclein, which is a main component of the Lewy bodies. Studies have shown that the content of ARTS and α-synuclein in the substantia nigra of PD patients is increased by more than 10-fold [68]. Studies have also found that overexpression of *Drosophila* ARTS-homologous genes can destroy the integrity of DA neurons in age-dependent dorsal clusters, and that this can be suppressed by increasing parkin co-expression. Conversely, it can also be enhanced by reducing the expression of parkin. This indicates that ARTS accumulation is toxic to DA neurons [69,70]. The *ARTS* gene is known to be a responsive target gene of p53. p53 binds to reactive DNA elements on the *ARTS* promoter and promotes its transcription. Inducers of p53 can increase the expression of ARTS, and the blocking of p53 can diminish the expression of ARTS under various stress conditions. These findings indicate that ARTS and p53 act synergistically on apoptosis involving mitochondria [71].

XIAP is a cell survival regulator and the most effective inhibitor of intracellular caspase. The typical apoptotic pathway includes the release of cytochrome *c* and the activation of caspase 9, caspase 7, and caspase 3 in sequence, which causes PARP cleavage and leads to apoptosis [15]. XIAP can inhibit the formation of functional dimers of caspase 9 in apoptotic bodies through the third BIR domain. At the same time, the second BIR domain can block the active sites of activated caspase 3 and caspase 7, thus preventing apoptosis. Studies have shown that specific gene transduction of XIAP in DA neurons can improve neurotoxicity and behavioral damage in MPTP-exposed PD models [72]. A recent study showed that XIAP can be bound by the E3 ligase RING-finger protein 166 (RNF166) and undergo ubiquitination-dependent degradation. 6-OHDA treatment can enhance the expression of RNF166, thus accelerating the activation of caspase and the death of neurons. The down-regulation of RNF166 in cells can activate XIAP and alleviate 6-OHDA-induced cell death [73].

ARTS is known to inhibit XIAP activity, thereby promoting caspase activation [18]. In 6-OHDA-exposed neuronal cells, the level of ARTS will increase. Overexpression of parkin can reduce the level of ARTS and improve apoptosis caused by 6-OHDA [74]. In a healthy brain, ARTS can be degraded by parkin’s ubiquitination to maintain low levels and avoid caspase activation. However, in nerve cells containing low or mutated parkin, the activity of parkin to degrade ARTS is reduced or abolished. Finally, XIAP is inhibited by the binding of ARTS, which is sufficient to promote the activation of caspase 9, 7, and 3.

Our data are consistent with these results. 6-OHDA-exposed SH-SY5Y cells displayed augmented expression of ARTS, but reduced expression of XIAP. However, pretreatment with PMN could reverse this result. PMN may promote the ubiquitination and degradation of ARTS by inducing parkin expression. Low levels of ARTS are expected to reduce XIAP inhibition and caspase activation and alleviate the apoptosis caused by 6-OHDA. In addition, we showed that the use of RNAi to down-regulate the expression of parkin could significantly abolish the ability of PMN to reverse 6-OHDA-induced ARTS expression, XIAP inhibition, and caspase activation.

ARTS is also known to be an modulator of autophagy. Overexpression of ARTS reduces autophagy activity [19]. XIAP can be used as a ubiquitin E3 ligase to regulate proteasome activity [16], and it can also upregulate autophagy activity [17]. As for 6-OHDA exposure, α-synuclein-overexpressing SH-SY5Y cells showed augmented levels of ARTS, but lessened levels of XIAP, and pretreatment with PMN could reverse this result. The resulting low level of ARTS could alleviate the damage to nerve cells caused by overexpression of α-synuclein. We also found that the use of RNAi to down-regulate the expression of parkin could considerably abolish the ability of PMN to promote the UPS and autophagy and reduced the accumulation of α-synuclein. In short, PMN decreases ARTS-mediated degradation of XIAP by modulating the PINK1/Parkin pathway, thereby ameliorating 6-hydroxydopamine toxicity and α-synuclein accumulation in PD models of *C. elegans* and SH-SY5Y cell lines. Interestingly, studies have shown that XIAP regulates the level of ARTS by acting as a ubiquitin ligase, thereby providing a potential feedback mechanism to prevent harmful cell apoptosis [75].

The current view of PD comprehends the concept that α-synuclein aggregates can spread from neuron-to-neuron in a prionlike fashion from the peripheral nervous system to the brain, via the enteric nervous system [76] or sensory nervous system [77,78]. PMN may also decrease α-synuclein in the peripheral nervous system, enteric nervous system or sensory nervous system by activating UPS and autophagy and downregulate the transneuronal spread of α-synuclein.

In this study, we used *C. elegans* and SH-SY5Y models to verify the effectiveness of PMN in its neuroprotection, but the concentration of PMN used in both models does not reflect the translational correlation with the mammalian model. However, PMN has proven its anti-inflammatory function on lipopolysaccharide-induced acute lung injury mice model, bleomycin-induced acute lung injury rat model, osteoarthritis mice model, and dinitrochlorobenzene-induced atopic dermatitis mice model [22,23,79,80]. Mice were treated with PMN once a day at a dose of 5 mg/kg for 8 weeks. The results showed no obvious toxicity to mice or rats. In addition, the pharmacokinetics, tissue distribution and excretion of PMN on rat model have also been reported [81]. The above information can provide us with a reference for translational research.

Although orthologous gene of *SNCA* and associated neurodegeneration are not found in *C. elegans*, transgenic worms produced by overexpressing wild-type or mutant human α-synuclein (A53T or A30P) showed degeneration of DA neurons together with loss of the basal slowing response [82]. Therefore, worms can still be used as a platform for preliminary drug development against synucleinopathies.

In conclusion, our experimental data demonstrated that PMN can significantly improve the neurotoxicity induced by 6-OHDA and the accumulation of α-synuclein in a PD model, and may have considerable therapeutic applications in the future. By enhancing the PINK1/parkin pathway, PMN can significantly lessen the expression of ARTS and then promote a rise in XIAP to resist apoptosis and activate the UPS and autophagy. Since *C. elegans* does not have the structure of a mammalian brain, and SH-SY5Y cells are not true DA neurons, in follow-up studies, we will use a unilateral 6-OHDA lesion mouse model, human A53T α-synuclein-overexpressing transgenic mice, and DA neurons derived from iPS cells of PD patient to further evaluate the effectiveness of PMN, especially in terms of pharmacokinetics. Finally, the causes of PD are complex, and mitochondrial dysfunction and chronic inflammation are also important influencing factors. In the future, we hope to use NGS and molecular docking technology to study the improvement effects of PMN on mitochondrial dynamics, mitophagy, and α-synuclein-induced inflammasome activity of microglia. In an aging society, it is important and urgent to establish an effective treatment for PD. PMN may provide an opportunity that is worthy of further research.

## 4. Materials and Methods

### 4.1. Chemicals, C. elegans Strains and Synchronization

Synthesized PMN (mol. wt. 429.64, 98% purity) was purchased from Rainbow Biotechnology Co. Ltd. (Shilin, Taipei, Taiwan) and dissolved in DMSO as a stock solution (1 M). Other chemicals and culture media were acquired from Sigma-Aldrich (St. Louis, MO, USA) unless otherwise stated. Wild-type Bristol N2 *C. elegans*, transgenic BZ555 strain (Pdat-1::GFP), transgenic N5901 strain (Punc-54::α-Syn::YFP), transgenic DA2123 strain (Plgg-1:: GFP::lgg-1), and *Escherichia coli* strains OP50 and HT115 were obtained from the Caenorhabditis Genetics Center (University of Minnesota, Saint Paul, MN, USA). The general maintenance and synchronization of the worm were carried out using the previously described method [31]. All worms were cultured at 20 °C.

### 4.2. Food Clearance Assay for Worms

To determine the appropriate treatment concentration of PMN that did not affect the growth of worms, we used the food clearance assay as previously described [83]. First, the PMN was diluted into S-medium to the indicated concentration, and then OP50 *E. coli* that had been allowed to grow overnight was evenly dispersed in PMN/S-medium with an optical density (OD) of 6.6. We loaded 50 µL of PMN//OP50/S-medium (OD = 0.6) to each well of a 96-well plate, and then added about 10 µL of medium to contain 20 L1 worms, and finally sealed the plate with a cover plate to prevent evaporation. Once per day, we measured the OD of the culture at 595 nm using a SpectraMax M2 Microplate Reader (Molecular Devices, Silicon Valley, CA, USA) for a total of 6 days. Before OD was measured, each plate was placed on a plate shaker and shaken for 30 s. After the measurement, the number and type of worms in each well were observed simultaneously under the microscope.

### 4.3. 6-OHDA Exposure and PMN Pretreatment of Worms

We exposed the worms to 6-OHDA according to the previously described method to cause DA neuronal degeneration [83]. First, L1 worms were transferred to OP50/NGM medium without or with different concentrations of PMN and cultured to L3 stage (24 h), and then exposed to 6-OHDA solution (50 mM, containing 10 mM ascorbic acid) for 1 h. During this period, the tube wall was tapped every 10 min to suspend the worms. After treatment, the worms were washed with M9 buffer and transferred to OP50/NGM/5-fluoro-2′-deoxyuridine, 2′-deoxy-5-fluorouridine (FUDR, 0.04 mg/mL) to reduce the generation of offspring. The worms were cultivated for 3 days until they reached the adult stage and were then used in various analyses.

### 4.4. Quantification of DA Neuron Degeneration in Worms

We used the previously described method to quantitatively analyze the DA neuronal degeneration of the BZ555 worm [83]. The reduction in the GFP signal of the DA neurons represents degeneration of the neurons. The worms were washed 3 times with M9 buffer, and then placed on the agar pad (2%) of a glass slide, anesthetized with sodium azide (100 mM), and then covered with a cover glass. The fluorescence of DA neurons in the head of the worm was imaged with Zeiss Axio Imager A1 fluorescence microscope (Carl Zeiss MicroImaging GmbH, Göttingen, Germany), and ImageJ software (National Institutes of Health, Bethesda, MD, USA) was used to determine fluorescence intensity. In addition, if the dendrites of the DA neurons of a worm showed bubbles or were absent, we recorded that the worm was positive for neurodegeneration of DA neurons.

### 4.5. Food Sensitivity Behavior Test in Worms

We used the previously described food-sensing behavior test to evaluate the function of DA neurons in worms [83]. To prepare a measurement plate, *E. coli* were spread in a ring on a 9 cm NGM plate with an inner diameter of 1 cm and an outer diameter of 8 cm, and the plates were cultured overnight. N2 adult worms of different treatments were washed with M9 buffer and then dropped into the center of the plate. Five minutes after the transfer, the number of S-shaped movements of each worm on the bacteria-free lawn and the bacterial lawn were measured three times at an interval of 20 s. The slowing rate = (the number of S-shaped movements in the bacteria-free lawn—the number of S-shaped movements in the bacterial lawn)/the number of S-shaped movements in the bacteria-free lawn. In all analyses, each group was anonymously labeled, so that the experimenter was unaware of the treatment of the worm. The average slowing rate of 50 worms was calculated for each group.

### 4.6. Lifespan Test in Worms

We use the previously described method for the worm lifespan test [83]. L3 stage N2 worms of different treatment groups were transferred to a plate (containing FUDR) for lifespan analysis, and a new plate was replaced every 3 days until all worms died. The number of surviving worms was counted daily. If a worm did not respond to repeated touches of the platinum picker, it was counted as dead. The analysis excluded worms that were removed from the wall and died as a result of dehydration. The survival curve was displayed by using Kaplan–Meier estimator and SPSS software (IBM, Armonk, NY, USA).

### 4.7. Quantification of Accumulation of Human α-Synuclein in Worm Muscle Cells

We used the previously described method to quantify the accumulation of α-synuclein in the NL5901 worm [83]. The synchronized L1 stage worms were cultured on OP50/NGM plates with or without PMN for 1 day. The worms were then transferred to OP50/NGM/FUDR plates with or without PMN, incubated for 3 days, and then washed with M9 buffer three times. The YFP signal of the worm reflects the accumulation of α-synuclein. The measurement and quantification method of fluorescence intensity is as described in 4.4.

### 4.8. Analysis of Protein Expression in Worms

We used the previously described method to extract proteins from the worms and perform Western blot analysis [31]. Protein was extracted from frozen worm pellets using Fastprep24 (MP Biomedicals LLC, Solon, OH, USA) and PBS containing protease inhibitors. The extract was boiled with sample buffer containing sodium dodecyl sulfate (SDS) for 10 min and separated by 10% SDS-polyacrylamide gel electrophoresis (SDS-PAGE). The proteins were then transferred to a polyvinylidene fluoride (PVDF) membrane. After reacting with the primary antibody overnight, the position and intensity of human α-synuclein were determined by use of horseradish peroxidase (HRP)-conjugated secondary antibody (PerkinElmer Inc., Boston, MA, USA) and the Amersham enhanced chemiluminescence system (Amersham Biosciences, Piscataway, NJ, USA) and BioSpectrum imaging system (UVP, Upland, CA, United States). Human α-synuclein monoclonal antibody (sc-12767) and β-actin (sc-47778) antibody were purchased from Santa Cruz Biotechnology (Santa Cruz, CA, USA).

### 4.9. Determination of Reactive Oxygen Species Content in Worms

We used the 2′,7′-dichlorodihydrofluorescein diacetate (H2DCFDA) analysis method to quantify the ROS content in worms [31]. Thirty worms were washed 3 times with M9 buffer and transferred to a 96-well plate with 150 μL PBS per well. We then added 50 μL of H2DCFDA (150 μM in PBS) and measured fluorescence at 20 °C using a SpectraMax M2 Microplate Reader (Molecular Devices, Silicon Valley, CA, USA) (λex = 485; λem = 520 nm). Fluorescence was measured every 15 min for a total of 150 min.

### 4.10. Total RNA Extraction and qPCR of Worms

We used the previously described method, using TRIzol reagent (Invitrogen, Carlsbad, CA, USA) and glass beads, to extract total worm RNA and then performed qPCR analysis [31]. The SuperScript one-step RT-PCR kit (Invitrogen), SYBR Green I Master kit (Roche Diagnostics, Indianapolis, IN, USA), and ABI StepOnePlus system (Applied Biosystems, Inc., Foster City, CA, USA) were used according to the manufacturer’s instructions. Table 1 lists the primer pairs for this experiment [31]. The comparison 2−^ΔΔCt^ method was used for analysis and the endogenous control was calculated by using *act-1* expression as the fold difference.

### 4.11. Determination of the Proteasome Activity of Worms

We used the previously described method to determine the proteasome activity (chymotrypsinlike activity) in worms [31]. Worms were first lysed by using a Precellys 24 homogenizer and proteasome activity assay buffer [50 mM Tris-HCl (pH = 7.5), 250 mM sucrose, 2 mM ATP, 5 mM MgCl_2_, 1 mM dithiothreitol, and 0.5 mM EDTA]. The lysate was then centrifuged at 10,000× *g* at 4 °C for 15 min. For each sample, 25 μg of total lysate was added to each well of a 96-well microtiter plate, and then the fluorescent substrate Suc-Leu-Leu-Val-Tyr-AMC (Sigma-Aldrich, St. Louis, MO, USA) was added. After the plates were incubated for 1 h at 25 °C, the fluorescence was measured with a SpectraMax M2 Microplate Reader (Molecular Devices, Silicon Valley, CA, USA) (λex = 380; λem = 460 nm).

### 4.12. Determination of the Autophagy Activity of Worms

We used the previously described method to observe the autophagy activity in transgenic DA2123 worms (which have a GFP-tagged LGG-1 regulated by the *lgg-1* promoter) [31]. The worms were washed 3 times with M9 buffer, and the LGG-1::GFP-positive puncta area in the outer epidermal seam cells was observed by using a fluorescence microscope. The positive punctate areas of at least 20 seam cells per worm were counted. At least 50 worms were counted in each group.

### 4.13. RNA Interference of Worm

We used the previously described method to perform RNA interference on worms fed *E. coli* that can express *pdr-1* siRNA [31]. RNase III-resistant *E. coli* (HT115(DE3)) has a *pdr-1*-specific double-stranded RNA expression plasmid (L4440) (Open Biosystems, Huntsville, AL, USA) that can be induced by IPTG. Feeding this *E. coli* to worms can target endogenous *pdr-1* mRNA and promote its specific degradation. In the 6-OHDA-exposed BZ555 model, the L1 worms were transferred to *pdr-1* RNAi/NGM plates with or without PMN and grown to the L3 stage. Next, the worms were exposed to 6-OHDA for 1 h and then cultured in *pdr-1* RNAi/NGM/FUDR plates with or without PMN for 3 days until analyzed. In the transgenic NL5901 model, L1 worms were cultured to *pdr-1 RNAi*/NGM plates with or without PMN for 1 day, and then transferred to *pdr-1 RNAi*/NGM/FUDR plates with or without PMN and cultured for 4 days until analyzed.

### 4.14. PMN Pretreatment and 6-OHDA Exposure of SH-SY5Y Cell Line

Human neuroblastoma SH-SY5Y cells (20th generation) were a generous gift from Chia-Wen Tsai (China Medical University, Taichung, Taiwan). We used the previously described method for cell culture [31]. Cells (1.2 × 10^6^) were inoculated on a 35 mm culture dish containing PMN at the specified concentration, allowed to incubate for 24 h, and then exposed to 100 μM 6-OHDA for 12 h (for Western blotting analysis) or 18 h (for MTT (3-(4,5-dimethylthiazol-2-yl)-2,5-diphenyltetrazolium bromide) assay, mitochondrial membrane potential (MMP) measurement, Hoechst 33258 staining, and Annexin-V FITC and PI staining). DMEM, penicillin-streptomycin, trypsin-EDTA, and fetal bovine serum were purchased from Gibco, ThermoFisher Scientific (Waltham, MA, USA).

### 4.15. Preparation of SH-SY5Y Cell Line Transiently Overexpressing α-Synuclein

To obtain the SH-SY5Y cell line overexpressing α-synuclein, we first synthesized the SNCA coding sequence (Accession numbers: NM_000345, Genewiz Inc., South Plainfield, NJ, USA). Then, the sequence was amplified by PCR and cloned into pcDNA3.1(+)-Myc vector (Invitrogen, ThermoFisher Scientific, Carlsbad, CA, USA) using *Nhe*I and *Apa*I sites via restriction enzymes (New England Biolabs, Beverly, MA, USA). Finally, according to the manufacturer’s instructions, Lipofectamine 2000 reagent (Invitrogen) was used to transfect the vector into the SH-SY5Y cell line. Empty pcDNA3.1(+) plasmids were used as the control group. The transfected cells were selected by use of G418 (1.5 mg/mL).

### 4.16. Immunofluorescence Staining of SH-SY5Y Cells

We used the previously described method for immunofluorescence staining [31]. Briefly, cellular samples grown on poly-L-lysine-coated coverslips were washed and fixed with 4% paraformaldehyde at room temperature for 10 min and then incubated with 0.2% Triton X-100 for 10 min. Next, the samples were soaked in a solution containing 1% BSA and 22.52 mg/mL glycine (dissolved in PBST (PBS + 0.1% Tween 20)) for 30 min. Primary antibody was added and allowed to react overnight at 4 °C. The next day, the sample was washed and placed in PBST containing 1% BSA. Goat anti-mouse IgG secondary antibody-Alexa Fluor 488 conjugate (purchased from Invitrogen) was added at the same time, and the sample was reacted at 25 °C for 1 h. Finally, the sample was washed, the nuclei were stained with DAPI, and the fluorescence was detected using a fluorescence microscope. Myc antibody was purchased from Santa Cruz Biotechnology, Inc. (Santa Cruz, CA, USA).

### 4.17. Cytotoxicity Analysis of PMN

We used the previously described MTT method to determine cell viability [31]. The SH-SY5Y cells were washed and replaced with fresh medium, and then MTT (5 mg/mL) was added and incubated at 37 °C for 2 h. Next, after washing, the formazan crystals were dissolved with isopropanol and the absorbance was measured at 570 nm using a Microplate Reader (Molecular Devices, Silicon Valley, CA, USA).

### 4.18. Western Blot Analysis of the SH-SY5Y Cell Line

For Western blot analysis of SH-SY5Y cells, we used the previously described method [31]. SH-SY5Y cells were washed twice with cold PBS and then collected in lysis buffer (25 mM Tris-HCl, 150 mM NaCl, 1% Triton X-100, 10% glycerol, 2 mM EDTA, 1 mM PMSF, 1 μg /mL leupeptin, 1 μg/mL aprotinin, and phosphatase inhibitor). Whole cell lysates were centrifuged at 14,000× *g* for 20 min at 4 °C. The protein concentration was measured with Coomassie plus protein assay reagent kit (Pierce, Rockford, IL, USA). The cell protein (50 μg) was then analyzed on 7.5%, 10%, or 12.5% SDS-PAGE gels, as shown in 4.8. Caspase 9 (#9508), cleaved caspase 9 (#20750), caspase 7 (#12827), cleaved caspase 7 (#8438), caspase 3 (#9662), cleaved caspase 3 (#9661), poly-ADP ribose polymerase (PARP) (#9542), cleaved PARP (#5625), mTOR (#2983), p-mTOR (#5536), and Atg7 (#8558) antibodies were from Cell Signaling Technology (Beverly, MA, USA). Monoclonal antibodies to PINK1 (sc-518052), parkin (sc-32282), ARTS, XIAP (sc-55550), PI3 kinase p100 (sc-365404), LC3 (sc-398822), and β-tubulin (sc-166729) were from Santa Cruz Biotechnology, Inc. (Santa Cruz, CA, USA). ARTS antibody (PA5-82767) was from Invitrogen ThermoFisher Scientific. HRP goat anti-rabbit and HRP goat anti-mouse secondary antibodies were from PerkinElmer, Inc. (Boston, MA, USA).

### 4.19. Measurement of Mitochondrial Membrane Potential in SH-SY5Y Cell Line

For the measurement of MMP of the SH-SY5Y cell line, we used the previously described 3,3’-dihexyloxacarbocyanine iodide (DiCO6) method [31]. The cells were washed with PBS and replaced with fresh medium and exposed to DiCO6 dye (1 μM). Thirty minutes later, the changes in MMP were recorded by use of a fluorescence microscope (green fluorescence) and the fluorescence intensity of the image was quantified by using ImageJ software (National Institutes of Health).

### 4.20. Staining of Hoechst 33258 in SH-SY5Y Cell Line

For Hoechst 33258 nuclear staining of the SH-SY5Y cell line, we used the previously described method [31]. The cells were washed with PBS and replaced with fresh medium. Cells were then stained with Hoechst 33258 (5 μg/mL) in the dark at 25 °C for 1 h, and changes in chromosome morphology (blue fluorescence) were recorded by using a fluorescence microscope.

### 4.21. Apoptosis Assay by Flow Cytometry

We used the FITC Annexin-V Apoptosis Detection Kit I (BD Biosciences Pharmingen, San Diego, CA, USA) to perform apoptosis analysis according to the manufacturer’s instructions. The cells were collected by trypsinization, washed three times with PBS, and centrifuged at 1500× *g* for 5 min at room temperature. Next, the cells were resuspended in 100 μL of 1× binding buffer (10 mM HEPES/NaOH (pH 7.4), 140 mM NaCl, and 2.5 mM CaCl_2_), and then annexin-V FITC and PI were added and the cells stained for 15 min in the dark. Finally, 400 μL of 1× binding buffer was added and the apoptosis rate was immediately analyzed using a BD LSRII flow cytometer (Becton Dickinson, Heidelberg, Germany). The cell collection gate for each sample contains at least 10,000 events to be collected. Among them, Q2 is a late apoptotic cell, Q4 is an early apoptotic cell, Q3 is a live cell, and Q1 is a dead cell. Apoptosis rate = (Q2 + Q4)/(Q1 + Q2 + Q3 + Q4) × 100%.

### 4.22. RNA Interference of SH-SY5Y Cell Line

The sequence of small RNA interference (siRNA) of parkin is as follows: 5′-UUCGCAGGUGACUUUCCUCUGGUCA-3′ (Tri-I Biotech Inc, Taipei, Taiwan). We used Lipofectamine 2000 reagent (Invitrogen) for siRNA transfection according to the manufacturer’s instructions. In the 6-OHDA exposure experiment, cells were transfected with control siRNA or parkin siRNA for 24 h, then pretreated with 1 μM PMN for 24 h, and finally treated with 100 μM 6-OHDA for 12 h (Western blotting) or 18 h (other analyses). In the SH-SY5Y cell line overexpressing α-synuclein (48 h after transfection), the cells were transfected with control siRNA or parkin siRNA for 24 h, and then treated with 1 mM PMN for 24 h.

### 4.23. Determination of Proteasome Activity in SH-SY5Y Cell Line

We used the previously described method to measure proteasome activity (chymotrypsinlike activity) in the SH-SY5Y cell line [31]. Cell lysate was incubated with enzyme substrate (Suc-Leu-Leu-Val-Tyr-AMC (Sigma-Aldrich, St. Louis, MO, USA)) at 25 °C for 1 h, and the Microplate Reader (Molecular Devices, Silicon Valley) was used to detect the fluorescence intensity corresponding to the proteasome chymotrypsinlike activity (λex = 380; λem = 460 nm).

### 4.24. Acidic Vesicular Organelle Staining in SH-SY5Y Cell Line

We used a method described previously to perform acidic vesicular organelle staining in the SH-SY5Y cell line [31]. After removal of the medium, the cells were washed with PBS, and incubated with acridine orange hydrochloride solution (0.5 μg/mL) at 37 °C for 10 min in the dark. After incubation, the formation of acidic vesicular organelles (red color) was detected under a fluorescence microscope. The fluorescence intensity was quantitatively analyzed by using ImageJ software (National Institutes of Health).

### 4.25. Determination of Autophilic Activity in SH-SY5Y Cell Line

We used the Autophagy Assay Kit (Sigma-Aldrich) according to the manufacturer’s instructions to determine the autophilic activity of the SH-SY5Y cell line. Cells were cultured in 96-well plates to optimal density (1 × 10^4^ cells/well) for the various treatment. The medium was removed from the cells and 100 µL of the autophagosome detection reagent working solution was added (diluting 500× Autophagosome Detection Reagent in the Stain buffer) to each well. Cells were incubated for 1 h and then washed with the Wash Buffer three times. We measured fluorescence intensity (λex = 360; λem = 520 nm) using a Zeiss Axio Imager A1 fluorescence microscope (Carl Zeiss), microplate reader (Molecular Devices), or BD LSRII flow cytometry (Becton Dickinson).

### 4.26. Statistical Analysis

Statistical analysis was implemented using SAS software (SAS, Institute.Inc, Cary, NC, USA). Each experiment was performed at least three times. Data are expressed as mean ± standard deviation (SD). We determined statistical significance by employing one-way ANOVA and Tukey’s test. Two groups were compared by using Student’s *t*-test. *p* values < 0.05 were assumed to indicate statistical significance.

## Figures and Tables

**Table 1:** Primers for real time PCR [31].

Genes of *C. elegans* (Human)	Primer Sequences (5′-3′)	(Start→End) Size (bp)
Lrk-1 (LRRK1)	Forward: TTTCAACACCCAATCTCCAAC Reverse: TGATACTCGCTTGCCACAC	(1983→2092) 110
Pdr-1 (PRKN)	Forward: TGCTCGTCAACCTCTGTTC Reverse: TCACTTTCTCCTTCCCATCAC	(376→601) 226
Pink-1 (PINK1)	Forward: GAGACGATACCGACAAACAC Reverse: GGCATTTCCTCCAAGACTAAC	(882→1158) 277
Djr-1.1 (PARK7)	Forward: CGGATTAGATGGAGCCGAAC Reverse: ATCAGCCCACCAGACTCTAC	(111→305) 195
Djr-1.2 (PARK7)	Forward: GCTTTGATCCTTTTGCCACC Reverse: CTGCCAGTTTGCTACATCC	(19→247) 229
Vps-35 (VPS35)	Forward: AACTCTGCTCAAAACTACTCAC Reverse: CCACAACCTTCTTCCCATTC	(1953→2146) 194
Catp-6 (ATP13A3)	Forward: TCACACCATACCAACCTCC Reverse: GTTTCCAAGAGTCTTCAGAACC	(3092→3336) 245
Dnj-27 (DNAJC10)	Forward: TCCACTTATTGCTCACATTGTC Reverse: TCCACCATCAACTCCACATC	(427→635) 209

## Data Availability

All data used and analyzed during the current study are available from the corresponding author on reasonable request.
